# Comprehensive analysis of the mouse cytochrome P450 family responsible for omega-3 epoxidation of eicosapentaenoic acid

**DOI:** 10.1038/s41598-018-26325-4

**Published:** 2018-05-21

**Authors:** Yosuke Isobe, Mai Itagaki, Yuko Ito, Satoko Naoe, Kotoe Kojima, Mitsunori Ikeguchi, Makoto Arita

**Affiliations:** 1 Laboratory for Metabolomics, RIKEN Center for Integrative Medical Sciences (IMS) 1-7-22, Suehiro-cho, Tsurumi-ku, Yokohama, Kanagawa 230-0045 Japan; 2 Graduate School of Medical Life Science, Yokohama City University, 1-7-29, Suehiro-cho, Tsurumi-ku, Yokohama, Kanagawa 230-0045 Japan; 3 Molecular Profiling Research Center for Drug Discovery (molprof), National Institute of Advanced Industrial Science and Technology (AIST), 2-4-7 Aomi, Koto-ku, Tokyo, 135-0064 Japan; 4 Division of Physiological Chemistry and Metabolism, Keio University Faculty of Pharmacy, 1-5-30, Shibakoen, Minato-ku, Tokyo, 105-0011 Japan

## Abstract

Metabolites generated via oxygenation of the omega-3 double bond (omega-3 oxygenation) in eicosapentaenoic acid (EPA) have recently been identified as novel anti-inflammatory lipid mediators. Therefore, oxygenase(s) responsible for this metabolic pathway are of particular interest. We performed genome-wide screening of mouse cytochrome P450 (CYP) isoforms to explore enzymes involved in omega-3 oxygenation of EPA. As a result, 5 CYP isoforms (mouse Cyp1a2, 2c50, 4a12a, 4a12b, and 4f18) were selected and identified to confer omega-3 epoxidation of EPA to yield 17,18-epoxyeicosatetraenoic acid (17,18-EpETE). Stereoselective production of 17,18-EpETE by each CYP isoform was confirmed, and molecular modeling indicated that chiral differences stem from different EPA binding conformations in the catalytic domains of respective CYP enzymes.

## Introduction

Dietary omega-3 polyunsaturated fatty acids (PUFAs), such as eicosapentaenoic acid (EPA) and docosahexaenoic acid (DHA), exert a wide range of beneficial effects on human health^1–3^. However, the molecular mechanisms for the beneficial actions of omega-3 PUFAs remain poorly understood. It was conventionally thought that omega-3 PUFAs have a suppressive effect on the formation of bioactive lipid mediators derived from omega-6 arachidonic acid (AA), such as prostaglandins and leukotrienes^4^. However, recent studies have shown that they also serve as precursors of anti-inflammatory lipid mediators^5,6^. E-series resolvins are lipid mediators derived from EPA that actively dampen inflammation to maintain tissue homeostasis^7–11^. They are biosynthesized through several lipoxygenase pathways from a common precursor, 18-hydroxyeicosapentaenoic acid (18-HEPE). In addition to E-series resolvins, we recently found a novel EPA metabolic pathway through omega-3 epoxidation (*i.e*. 17,18-EpETE), and 12-OH-17,18-EpETE was identified as an anti-inflammatory metabolite^12^. 18-HEPE and 17,18-EpETE not only serve as precursors for E-series resolvins and 12-OH-17,18-EpETE, respectively, but they also elicit potent bioactivities when administered *in vivo*. For example, 18-HEPE prevents pressure overload–induced maladaptive cardiac remodeling^13^, and 17,18-EpETE attenuates the development of intestinal diarrhea in a murine food allergy model^14^. 17,18-EpETE also induces relaxation in human pulmonary artery and airway smooth muscles through the activation of calcium-activated potassium (BK) channels that reduce calcium sensitivity of the contractile apparatus^15^. Both 18-HEPE and 17,18-EpETE are generated from EPA through oxygenation of the omega-3 double bond, which distinguishes EPA from omega-6 AA. Therefore, oxygenation of the omega-3 double bond may be an important metabolic pathway that contributes to the unique biological properties of omega-3 PUFAs. Therefore, enzymes responsible for omega-3 oxygenation in biological systems are of particular interest.

The biosynthesis of PUFA-derived lipid mediators is mainly initiated by cyclooxygenase (COX), lipoxygenase (LOX), and cytochrome P450 (CYP) enzymes^16,17^. The COXs convert AA and EPA to prostaglandin (PG) H_2_ and PGH_3_, which are then further metabolized by other enzymes to various PGs, thromboxanes, and prostacyclins. Of interest, acetylation of COX-2 by aspirin blocks its ability to produce PGs, but the enzyme remains active *in situ* to generate 18*R*-HEPE from EPA^18^. LOXs produce several hydroperoxides that go on to form leukotrienes, hydroxylated fatty acids, and lipoxins. CYP enzymes are membrane-bound monooxygenases that share a conserved structural core with a reactive heme group at the active site. The mouse genome encodes more than a hundred CYP enzymes that catalyze metabolism of a diverse range of small molecules such as drugs, industrial chemicals, and xenobiotics. In addition, recent studies have shown that omega-3 PUFAs can be metabolized by CYP enzymes into a series of oxygenated metabolites^19–27^.

In the present study, we performed a genome-wide screening of mouse CYP enzymes to comprehensively determine the oxygenase activity that confers omega-3 oxygenation of EPA. We characterized candidate enzymes by determining regioselectivity in the oxygenation of AA, EPA, and DHA using liquid chromatography tandem mass spectrometry (LC-MS/MS) profiles^28^. Moreover, we investigated the stereochemistry of 17,18-EpETE production by CYP enzymes. To rationalize the observed stereoselectivity of EPA oxygenation by CYPs, molecular modeling of CYP–EPA interaction was also performed.

## Results

### Genome-wide Functional Screening of CYP Enzymes

**Figure 1:**
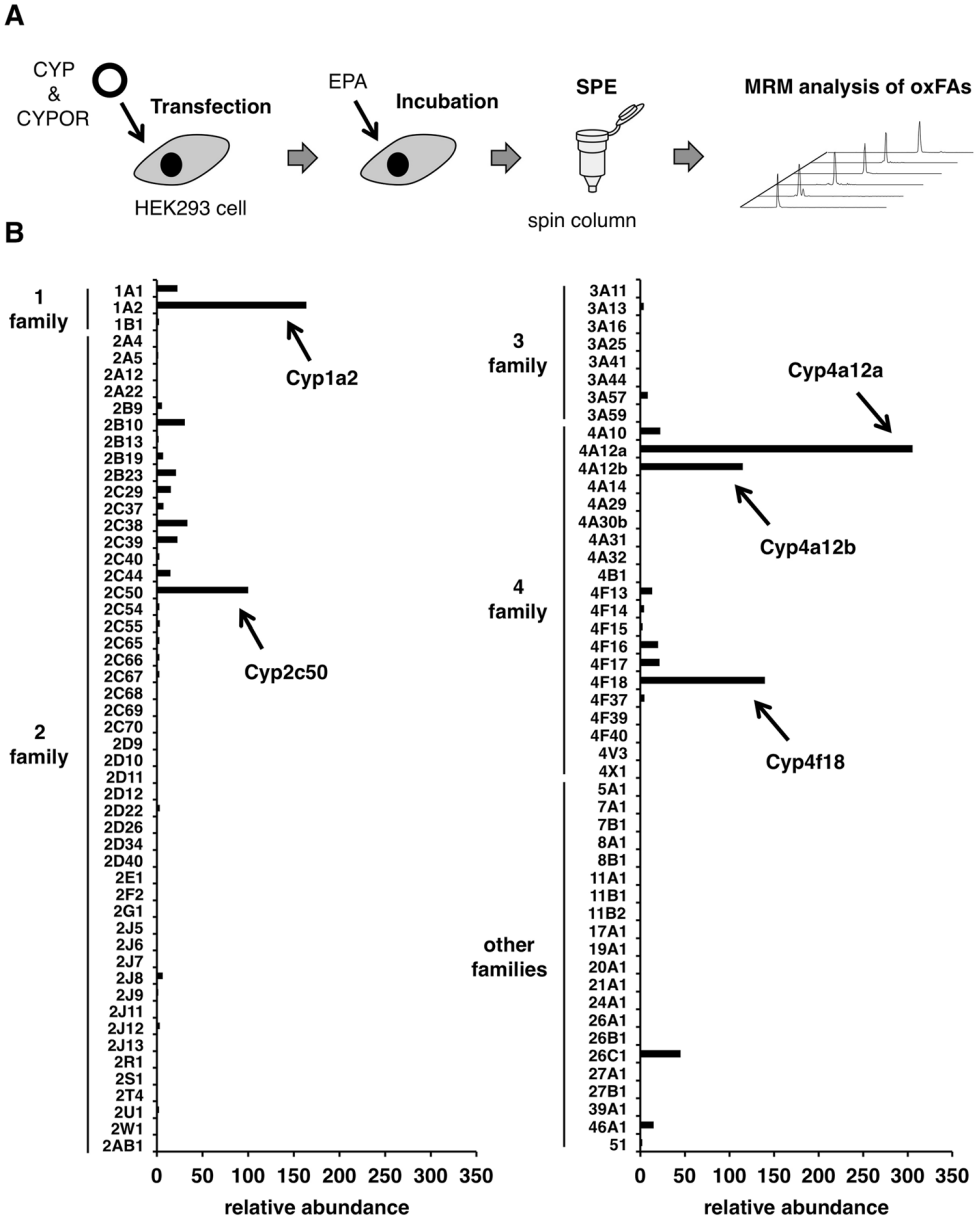
Genome-wide screening of mouse CYP enzymes. (**A**) Assay scheme. (**B**) Screening result showing production of 17,18-EpETE from EPA by HEK293 cells transiently transfected with the mouse CYP cDNA library. Data are represented as fold change relative to mock-transfected cells. MRM chromatograms are shown in Supplementary Fig. S2.

Previous studies have demonstrated that, among mouse CYPs, Cyp2c44, 4a12a, and 4a12b epoxidize the 17,18-olefinic bond of EPA to form 17,18-EpETE^21,25,26^. Several members of Cyp1a, 2c, 2j, and 4a subfamilies in human and rat have also been shown their activity to produce 17,18-EpETE^19,23–27^. In addition, it has recently been reported that human CYP2J2 metabolize omega-3 endocannabinoids such as eicosapentaenoyl ethanolamide (EA) to form 17,18-EpETE-EA^29^. However, there are no comprehensive studies to evaluate the PUFA-metabolizing activity of CYP enzymes. To this end, we conducted a genome-wide comprehensive screening of all mouse CYP isoforms. The procedure is as follows (Fig. 1A): (i) transfection with expression plasmids encoding mouse CYP and CYP oxidoreductase (CYPOR), (ii) incubation with PUFA for 1 h, resulting in the production of oxPUFAs, (iii) spin column-based high-throughput lipid extraction, (iv) high-sensitive and simultaneous identification and quantification of oxPUFAs using LC-MS/MS. We confirmed the protein expression of all FLAG-tagged CYP enzymes tested in this study (Supplementary Fig. S1). Using this system, we evaluated the EPA-oxygenating activity of mouse CYP isoforms. Among 101 CYPs tested, a total of 17 CYP-transfected cells produced 17,18-EpETE more than 10 fold as compared to mock-transfected cells (Fig. 1B and Supplementary Fig. S2). These include Cyp1a, 2c, and 4a family enzymes that are known to produce 17,18-EpETE in mice and/or other species. While we observed negligible production of 17,18-EpETE from cells expressing Cyp2j family enzymes, these cells metabolized AA to generate epoxygenated and/or hydroxylated products as consistent with previous reports (Supplementary Fig. S3)^30^, indicating that Cyp2j enzymes were functionally expressed. Among all CYPs tested, we selected top 5 candidates (*i.e*. Cyp1a2, 2c50, 4a12a, 4a12b, and 4f18) for further analysis.

### Metabolism of AA, EPA, and DHA by the candidate CYPs

**Figure 2:**
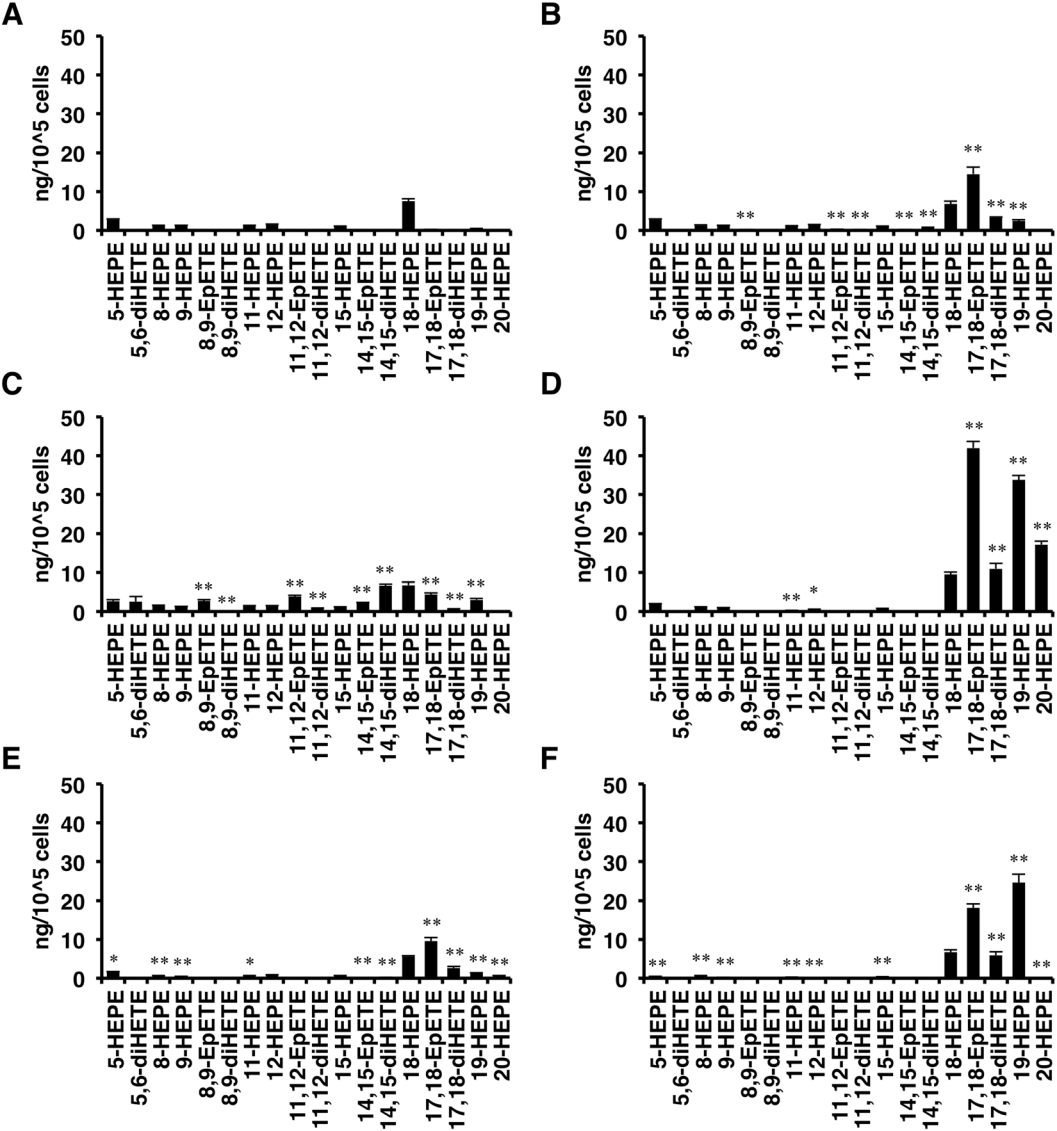
Regioselectivities of CYP isoforms for the oxidation of EPA. Amount of each metabolite formed from EPA by mock-transfected cells (**A**) or cells expressing Cyp1a2 (**B**), Cyp2c50 (**C**), Cyp4a12a (**D**), Cyp4a12b (**E**), or Cyp4f18 (**F**). Values represent the mean ± SEM; n = 3. *P < 0.05, **P < 0.01 when compared with mock-transfected cells.

We next investigated regioselectivity of EPA metabolism by the CYP isoforms. It should be noted that basal production of EPA metabolites was observed in mock-transfected cells (Fig. 2A). On the other hand, expression of CYP enzymes significantly increased the production of some metabolites which were characteristic of each CYP isoform (Fig. 2B–F). In Cyp1a2, epoxidation took place mainly at the omega-3 double bond when EPA was used as a substrate, as shown by dominant production of 17,18-EpETE and its corresponding diol 17,18-diHETE (Fig. 2B). On the other hand, Cyp2c50 was less regioselective but showed significant epoxygenase activity to produce EpETEs and/or their corresponding diols (Fig. 2C). Cyp4a12a and 4a12b produced 19-HEPE and 20-HEPE in addition to 17,18-EpETE and 17,18-diHETE (Fig. 2D,E). Cyp4f18 produced 17,18-EpETE, 17,18-diHETE, and 19-HEPE (Fig. 2F).

When AA or DHA was used as a substrate, the CYP isoforms exhibited substrate-dependent regioselectivity (Supplementary Table S1–5). Cyp1a2 acted mainly as an omega-3 epoxygenase to produce 17,18-EpETE or 19,20-EpDPE and corresponding diols (*i.e*. 17,18-diHETE or 19,20-diHDoPE, respectively) (Supplementary Table S1). In contrast, the same enzyme showed broad regioselectivity with AA. Cyp2c50 functioned as EPA epoxygenase with less regioselectivity, and also displayed epoxygenase activities for AA and DHA (Supplementary Table S2). Cyp4a12a and 4a12b showed relatively higher omega-hydroxylase activity converting EPA or AA to 20-HEPE or 20-HETE, respectively. However, their omega-hydroxylase activities were weaker when DHA was used as a substrate, and they predominantly produced 19,20-EpDPE (Supplementary Table S3 and S4). Cyp4f18 displayed regioselectivity in favor of producing 19-HEPE and 17,18-EpETE from EPA. However, Cyp4f18 showed increased omega-3 epoxygenase activities and weak omega-1 hydroxylase activities with DHA and mainly produced 19,20-EpDPE followed by 21-HDoHE (Supplementary Table S5).

### Stereoselective Epoxidation of the Omega-3 Double Bond of EPA

**Figure 3:**
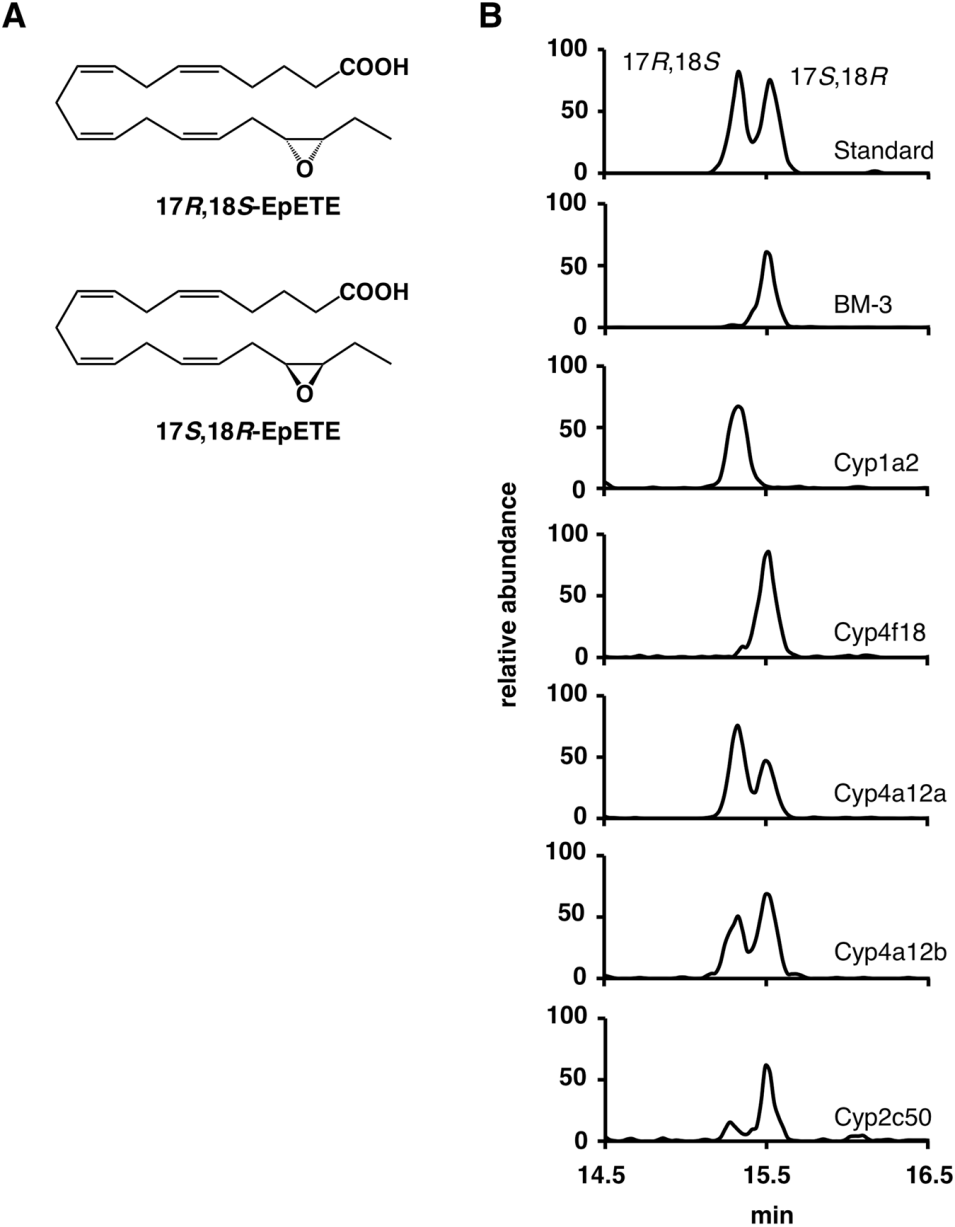
Stereochemistry of omega-3 epoxidation of EPA by candidate CYP isoforms. (**A**) Chemical structures of 17,18-EpETE stereoisomers. (**B**) Chiral analysis of 17,18-EpETE by LC-MS/MS. Representative chiral analysis illustrating the stereoselectivity of omega-3 epoxidation by BM-3 or mouse CYP isoforms as indicated.

In general, the stereochemistry of oxidized fatty acids is important for their biological activity. For example, among the 17,18-EpETE enantiomers shown in Fig. 3A, only the 17*R*,18*S*-enantiomer is effective on BK channels in rat cerebral arteries^31^. Therefore, we investigated the stereochemistry of the epoxidation reaction of EPA by candidate CYP isoforms. To evaluate the stereoselectivity of EPA epoxidation, products were analyzed by chiral-phase HPLC. Commercially available (±)17,18-EpETE resulted in two main peaks with retention times of 15.3 and 15.5 min (Fig. 3B). Since enantiomeric standards for 17,18-EpETE are currently unavailable, we identified these enantiomers from the well-known stereoselectivity of BM-3. BM-3, a bacterial CYP that closely resembles eukaryotic CYPs, is a highly regio- and stereoselective epoxygenase for EPA to yield 17*S*,18*R*-EpETE^32^. As expected, chiral-phase HPLC resulted in only one peak with a retention time of 15.5 min (Fig. 3B). Therefore, the use of 17,18-EpETE generated by BM-3 as a standard made it possible to identify the chiral isomers of 17,18-EpETE formed by the mouse CYPs tested.

As shown in Fig. 3B, Cyp1a2 displayed a high stereoselectivity in favor of producing 17*R*,18*S*-EpETE, which is the opposing enantiomer produced by BM-3. By contrast, Cyp4f18 generated an enantiomeric excess of 17*S*,18*R*-EpETE. As compared with these CYP isoforms, Cyp2c50, Cyp4a12a, and 4a12b displayed less stereoselectivity as described previously^21^. It should be noted that, in addition to 17,18-EpETE, the production of 17,18-diHETE was also observed by CYP-transfected cells (Fig. 2). This can be explained by the presence of active epoxide hydrolases, especially a soluble epoxide hydrolase (sEH), in HEK293 cells used as a host. Since epoxide hydrolases may function in a stereoselective manner^33^, we evaluated the stereoselectivities of candidate CYPs in the presence of sEH inhibitor, 12-[[(cyclohexylamino)carbonyl] amino]-dodecanoic acid (CUDA). While the ratio of 17,18-EpETE/17,18-diHETE was increased in the presence of CUDA, the chirality of 17,18-EpETE did not change (Supplementary Fig. S4). These results suggest that the chirality of 17,18-EpETE generated from CYP-transfected cells reflect the stereoselectivity of each CYP enzymes.

**Figure 4:**
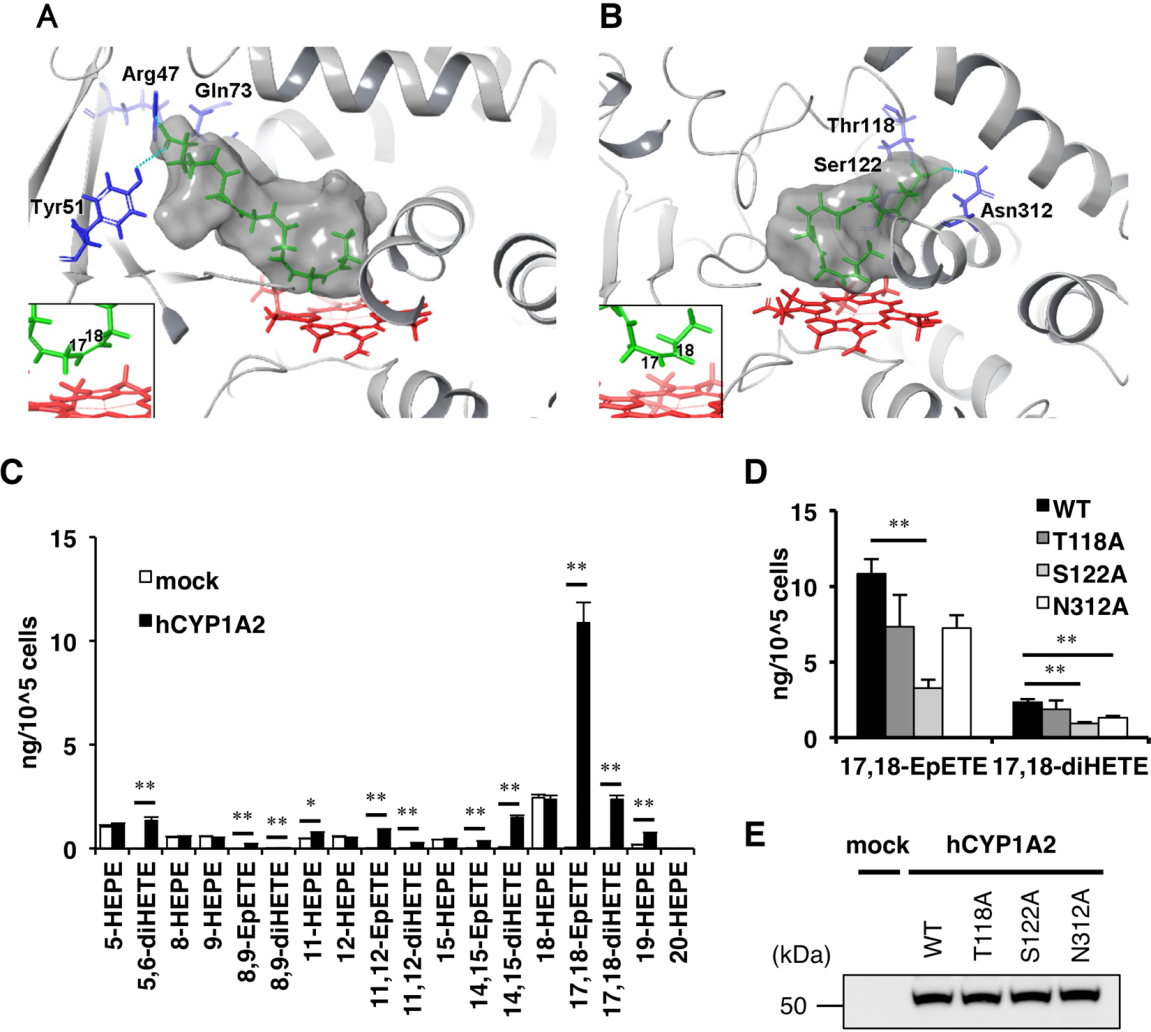
Structural analysis of BM-3 and human CYP1A2. Model of EPA bound to BM-3 (**A**) and CYP1A2 (**B**). The gray surface indicates the binding cavity identified by SiteMap. EPA and heme are shown in green and red, respectively. The residues interacting with the carboxyl group of EPA are colored blue. Located behind the binding cavity, Ser122 (**B**) is hidden by the surface pocket. Insets in A and B show the enlarged view of the conformation of the metabolized position of EPA. (**C**) Lipidomic profiles of EPA incubation products from HEK293 cells transiently transfected with mock (white bar) or human CYP1A2 (black bar). Values represent the mean ± SEM; n = 3. *P < 0.05, **P < 0.01. (**D**) Production of 17,18-EpETE and 17,18-diHETE by HEK293 cells transiently transfected with human CYP1A2 (WT, T118A, S122A, or N312A). Values represent the mean ± SEM; n = 3. **P < 0.01. (**E**) HEK293 cells were transiently transfected as indicated. Cell lysates were prepared 24 h after transfection, and expression of CYP1A2 was analyzed by western blotting.

We assumed that this chiral difference stems from the EPA binding conformation in the respective CYP enzymes. To investigate the EPA binding conformation, we adopted a theoretical approach combining docking and molecular dynamics (MD) simulations by using the structures of BM-3 and CYP1A2, which showed opposite stereoselectivities in omega-3 epoxidation of EPA. While several BM-3 crystal structures have been reported, the structure of mouse Cyp1a2 has not been determined. Thus, human CYP1A2 structures were used in the simulations. The amino acid sequence of human CYP1A2 is 74% identical to that of mouse Cyp1a2, which allows the human structure to be used as a substitution^34,35^. Figure 4A and B show the EPA binding poses obtained by theoretical predictions. When we compared the binding cavity between BM-3 and human CYP1A2 structures, their orientations relative to heme are completely different. The binding cavity in BM-3 is directed toward the near side of Fig. 4A. In contrast, the cavity in CYP1A2 is stretched out toward the far side of Fig. 4B. By necessity, the EPA binding poses between the two are different. In BM-3, Arg47, Tyr51, and Gln73 located on the near side are used in the interaction. In CYP1A2, Thr118, Ser122, and Asn312 located on the far side have hydrogen bonds with the carboxylate group of EPA. Similar EPA binding poses were observed in the re-docking simulations (Supplementary Figs S5 and S6, depicted in magenta). We next examined whether the candidate amino acid residues involved in ligand binding at the active site of CYP1A2 (*i.e*. Thr118, Ser122, and Asn312) are essential for its EPA-metabolizing activity. Similar to the mouse orthologue, human CYP1A2 mainly produced 17,18-EpETE from EPA, as described previously (Fig. 4C)^23,24^. Cells expressing the point mutant S122A, in which Ser122 was changed to Ala, had significantly reduced production of 17,18-EpETE and 17,18-diHETE from EPA (Fig. 4D). Expression of T118A or N312A also reduced production of 17,18-EpETE and 17,18-diHETE (Fig. 4D). These mutant proteins were expressed at almost the same level as the wild-type (WT) human CYP1A2 (Fig. 4E). These results suggest that these amino acid residues are involved in the recognition of EPA as a substrate.

## Discussion

We conducted a genome-wide screening to identify CYP enzymes that catalyze the omega-3 epoxidation of EPA. We investigated the formation of epoxidized and hydroxylated regioisomers of AA, EPA, and DHA by candidate CYPs as well as the stereoselectivity of omega-3 epoxide of EPA. As consistent with the present study, mouse Cyp4a12a and 4a12b were previously shown to display omega-3 epoxygenase activity with EPA to yield 17,18-EpETE^21,25^. Also EPA-metabolizing activity by human CYP1A2 was clearly demonstrated (Fig. 4C) as consistent with the previous reports^23,24^. These results provide validation for this method to screen for mouse and/or human CYP enzymes responsible for PUFA metabolism.

Cyp2c50 was identified as a primarily AA epoxygenase and produced all regioisomers of AA epoxides (EETs)^36^. As consistent with the previous findings, Cyp2c50 showed less regioselective but significant epoxygenase activities not only with AA but also with EPA (Fig. 2C and Supplementary Table S2). Cyp1a2 and 2c50 are mainly expressed in the liver, and it has previously been reported that omega-3 PUFA-rich diet increased EPA epoxides, especially 17,18-EpETE, in rat liver^25^. Therefore, Cyp1a2 and 2c50 are likely to be the major EPA-metabolizing enzymes in the liver. Cyp4a12a is highly expressed in mast cells (BioGPS, http://biogps.org/#goto=genereport&id=277753), and a recent study showed that mast cells produce omega-3 epoxide including 17,18-EpETE to promote their IgE-mediated activation^37^. Thus, Cyp4a12a may contribute to this process by locally producing 17,18-EpETE. Cyp4f18 has been identified as the omega-1 and omega-2 hydroxylase of leukotriene B_4_ (LTB_4_), which is a potent chemoattractant for myeloid cells such as polymorphonuclear leukocytes (PMNs)^38^. Cyp4f18 is expressed in PMNs, and this omega oxidation step results in LTB_4_ inactivation. A targeted deletion in the Cyp4f18 gene results in loss of LTB_4_ omega oxidation products in mouse PMNs^39^. On the other hand, there were no changes in LTB_4_-dependent chemotaxis between PMNs from wild type and Cyp4f18 knockout mice^40^. In addition, Cyp4f18 deficiency did not significantly increase inflammatory cell infiltration or injury following renal ischemia-reperfusion^39^. These results indicate that Cyp4f18 is not only an enzyme for inactivation of LTB_4_, but may also contribute to immunomodulatory function through the production of 17,18-EpETE.

Our investigation showed that the stereoselectivity of epoxidation of the omega-3 double bond of EPA differed between CYP isoforms. For example, when compared with BM-3, the opposite stereoselectivity was observed in Cyp1a2. As shown in Fig. 4A and B, the extended direction of the binding pocket is completely opposite between BM-3 and CYP1A2. While Arg47, Tyr51, and Gln73, located on the near side, are used for interaction with EPA in BM-3, Thr118, Ser122, and Asn312, located on the far side, have hydrogen bonds with the carboxylate group of EPA in CYP1A2. A previous modeling study using BM-3 and arachidonic acid showed that the fatty acid carboxylate was positioned within charge coupling distance of Arg47, which is located at the mouth of the active site in BM-3^32^. Thr118, Ser122, and Asn312 in CYP1A2 were important for metabolizing EPA (Fig. 4D). Throughout the entire MD simulation, the same interactions with the carboxylate of EPA in both BM-3 (Supplementary Figs S7 and S8A,B) and the CYP1A2 (Supplementary Figs S9 and S10A,B) were observed. Along with these interactions, the metabolized positions 17*S*,18*R* in BM-3 and 17*R*,18*S* in CYP1A2 remained exposed to Fe^2+^ of the heme during simulation (Supplementary Figs S8C,D and S10C,D). From these MD data, we speculate that in order for EPA to be bound in the appropriate enantiomeric conformation in respective CYPs, the carboxyl group of EPA needs to be in contact with specific residues in CYP1A2 and BM-3, which are located in completely different positions, as shown in Fig. 4A and B.

Previous studies have demonstrated a series of 17,18-EpETE’s bioactivities. In addition to anti-allergic effects and BK channel activation, treatment with 17,18-EpETE conferred significant, dose-dependent protection from laser-induced choroidal neovascularization (CNV) in an age-related macular degeneration (AMD) model^41^. 17,18-EpETE also significantly reduced palmitate-induced accumulation of lipids in adipocytes^42^. Therefore, cells expressing CYPs identified in this study may be involved in these biological processes by locally producing 17,18-EpETE. Further analysis of CYP-deficient mice should reveal the biological significance of EPA omega-3 epoxidation *in vivo*.

## Methods

### Chemicals

Fatty acids, oxidized fatty acids, and CUDA were purchased from Cayman Chemical. All LC/MS grade solvents were obtained from Sigma-Aldrich. Other chemicals were purchased from Wako Chemicals unless otherwise indicated.

### Molecular cloning of CYP isoforms

Mouse CYP isoforms were amplified by PCR with complementary DNA (cDNA) derived from mouse organs, or mouse cDNA collection of the FANTOM Consortium^43^ and OriGene. The products encoding mouse CYP isoforms were introduced into pCAGGS vectors^44^ with an N-terminal FLAG tag. cDNA that could not be cloned as described was chemically synthesized as gBlock Gene Fragments (Integrated DNA Technologies), and was inserted into pCAGGS vectors by homologous recombination using the In-Fusion HD Cloning Kit (Clontech) according to the manufacturer’s instructions.

### Genome-wide functional screening of CYP enzymes

HEK293 cells were seeded onto 24-well plates (1.0 × 10^5^ cells per well) in Dulbecco’s modified Eagle’s medium (DMEM) containing Penicillin-Streptomycin-Glutamine (PSG, Thermo Fisher Scientific) supplemented with 10% (v/v) fetal calf serum (FCS) in 5% CO_2_ at 37 °C and cultured to grow to 80% confluence for 24 h. After 24 h, cells were rinsed with PBS and conditioned medium was replaced with PSG-free DMEM supplemented with 10% (v/v) FCS.

HEK293 cells were transiently transfected with pCAGGS (an empty vector plasmid) or pCAGGS containing CYP cDNA (400 ng per well) and co-transfected with CYPOR (100 ng per well) using ViaFect Transfection Reagent (Promega). Transfected cells were cultured for 24 h under the same conditions as described. After 24 h, transfected cells were rinsed with Hanks’ balanced salt solution (HBSS, with Ca^2+^ and Mg^2+^, Thermo Fisher Scientific) supplemented with 0.1% (w/v) bovine serum albumin (BSA, Sigma-Aldrich), which was essentially fatty acid free, and treated with EPA, DHA, and AA (30 μM each) in HBSS. Treated cells were incubated at 37 °C for 1 h, and after incubation ice-cold methanol was added to stop the reaction. Culture supernatant and methanol were collected as samples.

### Flow cytometry analysis

For intracellular staining of FLAG-tagged CYPs, Foxp3 staining fixation/permeabilization buffer (eBioscience) was used according to the manufacturer’s protocol. Briefly, cells were treated with fixation/permeabilization buffer for 30 min and then stained with rat anti-FLAG antibody L5 (BioLegend) for 45 min and washed. The cells were incubated with a secondary antibody, Alexa Fluor 488–conjugated donkey anti-rat IgG (Thermo Fisher Scientific), for 30 min, and analyzed with a FACSCalibur (BD Biosciences) instrument. Data analysis and graphic output were performed using FlowJo software (Tomy Digital Biology).

### Sample extraction and LC-MS/MS-based lipidomics

oxPUFAs were purified from samples by solid-phase extraction using MonoSpin C18 (GL Sciences) with deuterium-labeled internal standards (15-HETE-d8, 14,15-EET-d11 and LTB_4_-d4) as reported previously^45^. Briefly, MonoSpin C18 columns were preconditioned with methanol and water, and samples were applied. Columns were then washed with water and hexane followed by the elution with methanol. Subsequent LC-MS/MS-based lipidomic analyses were performed using an HPLC system (UPLC, Waters) with a linear ion trap quadrupole mass spectrometer (QTRAP5500, Sciex) equipped with an Acquity UPLC BEH C_18_ column (1.0 mm × 150 mm × 1.7 µm; Waters) as reported previously^12,28^. Samples were eluted with a mobile phase composed of water/acetate (100:0.1, v/v) and acetonitrile/methanol (4:1, v/v) (73:27) for 5 min; ramped to 30:70 after 15 min, to 20:80 after 25 min, and held for 8 min; ramped to 0:100 after 35 min, and held for 10 min, with flow rates of 70 µl/min (0–30 min), 80 µl/min (30–33 min), and 100 µl/min (33–45 min). MS/MS analyses were conducted in negative ion mode, and fatty acid metabolites were identified and quantified by multiple reaction monitoring (MRM). MRM transitions, declustering potential, entrance potential, collision energy, collision cell exit potential, and retention time for all the analytes and internal standards are described in Supplementary Table S6. Compounds were quantified by using calibration curves, and recoveries were monitored using added deuterium-labeled internal standards. Stereoisomers were chirally separated with a CHIRALCEL OJ-3R column (4.6 mm × 150 mm × 3 μm; Daicel Corp). Samples were eluted with mobile phase composed of water/acetate (100:0.1, v/v) and acetonitrile/methanol (4:1, v/v) (50:50) for 5 min; ramped to 5:95 after 27.5 min, and held for 8 min with flow rates of 500 μl/min.

### Preparation of 17 S,18R-EpETE by Bacillus Megaterium BM-3

BM-3 was cloned into a pET-21a(+) vector (Novagen) and overexpressed as C-terminal His-tagged fusion proteins in *E. coli* BL21 (DE3) cells in LB medium. Expression was induced by addition of 1 mM isopropyl β-D-thiogalactopyranoside and cells were incubated at 20 °C overnight. The protein was purified using a HisTrap HP column (GE Healthcare) according to the manufacturer’s instructions and dialyzed against 50 mM Tris-HCl (pH 7.4) containing 1 mM EDTA, 10% glycerol.

The reaction mixture contained 30 μM EPA, 80 μg purified BM-3, and 50 mM 3-(N-morpho-lino)propanesulfonic acid (MOPS) buffer, pH 7.4. The reaction was started by addition of 50 mM NADPH. After 60 min, the reaction was stopped by addition of ice-cold methanol. Metabolites were then extracted as described.

### Western blotting

HEK293 cells transfected with pCAGGS or pCAGGS containing human CYP1A2 cDNA (WT, T118A, S122A, or N312A mutant) and co-transfected with human CYPOR as described were centrifuged and the cell pellet was resuspended in PBS. Cell lysates were prepared by brief sonication, and protein concentration was determined by a BCA Protein Assay Kit (Pierce). Proteins were separated by SDS-PAGE and transferred to PVDF membrane (Bio-Rad). The membranes were blocked with 5% (w/v) skim milk in TBS-T (10 mM Tris-HCl, pH 7.4, 150 mM NaCl, 0.05% (v/v) Tween 20), and blots were incubated with 1:1000 dilutions of monoclonal antibody to CYP1A2 (D15, Santa Cruz Biotechnology). After incubation with horseradish peroxidase-conjugated anti-mouse IgG antibody (GE Healthcare), CYP1A2 was detected by enhanced chemiluminescence using a Chemidoc Touch MP (Bio-Rad). An uncropped image is shown in Supplementary Fig. S11.

### Molecular modeling

Docking and MD simulations were performed using the Schrödinger software package (Schrödinger Release 2016-3, Schrödinger LLC, New York, 2016). The EPA molecule was docked into CYP structures using Glide version 7.2^46–48^. Subsequent MD simulations were conducted using DESMOND version 4.7^49,50^. Docking and MD simulations were performed with an OPLS-3 force field^51^. More details are provided as supplementary information.

### Statistical analysis

Results are expressed as the mean ± SEM. Differences between two groups were tested by the Student’s *t*-test. A significance level of p < 0.05 was used.

## Electronic supplementary material

Supplementary Information
