# Supplementary material for: Comprehensive analysis of the mouse cytochrome P450 family responsible for omega-3 epoxidation of eicosapentaenoic acid
Source: Sci Rep. 2018 May 21;8:7954. doi: 10.1038/s41598-018-26325-4 (PMC5962638; doi:10.1038/s41598-018-26325-4)
Supplement: Supplementary file 1 — Supplementary Information [file 41598_2018_26325_MOESM1_ESM.pdf]

Supplementary information:

Comprehensive analysis of the mouse cytochrome P450 family responsible for omega-3 epoxidation of eicosapentaenoic acid

Yosuke Isobe[1,2], Mai Itagaki[1,2], Yuko Ito[2,3], Satoko Naoe[1], Kotoe Kojima[1,2], Mitsunori Ikeguchi[2], and Makoto Arita[1,2,4]

Affiliations:

[1]Laboratory for Metabolomics, RIKEN Center for Integrative Medical Sciences (IMS) 1-7-22, Suehiro-cho, Tsurumi, Yokohama, Kanagawa, 230-0045, Japan

[2]Graduate School of Medical Life Science, Yokohama City University, 1-7-29, Suehiro-cho, Tsurumi, Yokohama, Kanagawa, 230-0045, Japan

[3]Molecular Profiling Research Center for Drug Discovery (molprof), National Institute of Advanced Industrial Science and Technology (AIST), 2-4-7 Aomi, Koto-ku, Tokyo 135-0064, Japan

[4]Division of Physiological Chemistry and Metabolism, Keio University Faculty of Pharmacy, 1-5-30, Shibakoen, Minato-ku, Tokyo, 105-0011, Japan

Supplementary methods

Molecular modeling

<Initial structures for simulations>

Crystal structures of the Cyp4f families used in these experiments have not been determined. Therefore, EPA docking simulations were performed using the BM-3 structure, which has the same properties as Cyp4f families in terms of positional and steric selectivity of EPA metabolism[1]. Many BM-3 structures have been determined. Compared with several BM-3 structures, the binding pocket shapes were fairly similar. Therefore, 4KPA (PDB cord)[2], which has good resolution and no mutations, was selected as showing common features to the binding pocket in BM-3 structures. Because the mouse Cyp1a2 structure used in the experiments is also unavailable, the human CYP1A2 2HI4 (PDB cord)[3] was used to predict the binding pose of EPA.

<The state of the iron atom in heme>

All cytochrome P450s contain heme as a cofactor, and the iron atom resides in the center of the heme molecule. During substrate oxidation, iron changes the ion states between Fe^{3+} and Fe^{2+} [4, 5]. In docking studies for both BM-3 and CYP1A2, we adopted the Fe^{2+} state. After the substrate binds to the Fe^{3+} heme binding site, cytochrome P450 takes a single electron from a reductase and changes to the reduced Fe^{2+} form, which is the state waiting for O_2 binding to the enzyme-substrate complex.

<Computational details of docking and MD simulations>

Docking simulations were performed using the Schrodinger software package (Schrödinger Release 2016-3, Schrödinger LLC, New York, 2016). In receptor preparation, the CYP structures were assigned charges, bound orders and hydrogens belonging to heavy atoms based on the Protein Preparation Wizard[6] in Maestro version 10.7 (Schrödinger Release 2016-3: Maestro, Schrödinger, LLC, New York, NY, 2016). Structures were then optimized at a neutral pH and minimized using the OPLS-3 force field[7]. For ligand preparation, we sketched the 2D molecular structure of EPA in a Maestro workspace and converted it to a 3D structure using the LigPrep module version 3.9 (Schrödinger Release 2016-3: LigPrep, Schrödinger, LLC, New York, NY, 2016). After converting to the 3D structure, a conformation search was performed to generate conformers, and low energy structures were searched for using the OPLS-3 force field[7].

Prepared EPA was docked into the BM-3 and CYP1A2 receptors individually using Glide version 7.2[8,9,10] (Schrödinger Release 2016-3: Glide, Schrödinger, LLC, New York, NY, 2016) and Glide induced-fit docking, respectively[11,12,13] (Schrödinger Release 2016-3: Induced Fit Docking protocol; Glide version 7.2. and Prime version 4.5, Schrödinger, LLC, New York, NY, 2016). Because the BM-3 crystal structure binds N-palmitoylglycine (long-chain fatty acid)[2], which is structurally similar to EPA, Glide could predict the EPA binding pose in the BM-3 pocket. By contrast, CYP1A2 is co-crystallized with α-naphthoflavone[3], which has a much different structure from EPA than N-palmitoylglycine (the BM-3 ligand). As a result, several amino acids in the binding cavity sterically hindered EPA binding and no docking poses were generated in Glide. In induced-fit docking of CYP1A2, the Phe226, Leu382 and Leu497 side chains were trimmed. Docking simulations in both BM-3 and CYP1A2 were performed using the OPLS-3 force field[7]. The grid box was placed on the center of the originally bound ligand. To obtain a docking pose consistent with the experimental data, a specific range (between 0.3 and 0.8) of scaling factors for van der Waals radius were used for the receptor and ligand.

Using the docking procedures described, only a few poses that were positionally and chirally appropriate for EPA metabolism were obtained for both BM-3 and CYP1A2 structures. This low success rate can be attributed to the pocket conformation of the docking receptors, which is basically the same as in the crystal structures and therefore not suitable for EPA binding and metabolism. To refine the obtained EPA-CYP complexes in Glide docking, the structures were subsequently subjected to MD simulations, which can optimize side chain and backbone atoms of proteins such that the receptor pocket is rearranged to fit the docked EPA. Calculations were conducted for 120 ns using DESMOND version 4.7[14,15] with an OPLS-3 force field[7]. In the MD simulations, the simple point charge (SPC) model[16] for water was used in a cubic box with periodic boundary conditions containing a 10 Å buffer distance to each dimension and the system was electrically neutralized at 0.15 M NaCl. Before performing the productive runs for 120 ns, the systems were relaxed by the default protocol for the NPT ensemble, which consists of five steps: (i) 100 ps NVT ensemble with Brownian dynamics at 10 K with solute restrained; (ii) 12 ps simulation in the NVT ensemble using a 10 K Berendsen thermostat with solute heavy atoms restrained; (iii) 12 ps simulation in the NPT ensemble using a 10 K Berendsen thermostat and a 1 atm Berendsen barostat with solute heavy atoms restrained; (iv) 12 ps NPT ensemble using a 300 K Berendsen thermostat and a 1 atm Berendsen barostat with solute heavy atoms restrained; and (v) 24 ps NPT ensemble using a 300 K Berendsen thermostat and a 1 atm Berendsen barostat with no restraints. In the 120 ns

productive runs, 300 K temperature and 1 atm pressure were maintained using the Nosé–Hoover thermostat[17] and Martyna–Tobias–Klein barostat algorithms[18,19]. The short-range electrostatic interactions were analyzed using a cut-off value of 9.0 Å. To treat long-range electrostatic interactions, the particle-mesh Ewald method was used in which a tolerance value of 1e-9 was set[20]. A multiple time step approach, RESPA, was employed where default values of 2.0, 2.0 and 6.0 fs were used for bonded, short-range nonbonded, and long-range nonbonded electrostatic interactions, respectively[21]. To enable the time step, the SHAKE algorithm was used[22].

In MD simulations, distance restraints were applied (k=3.0) between the metabolized position of EPA and the Fe^{2+} ion of the heme (C17-Fe^{2+} and C18-Fe^{2+}) because the pocket in the initial structure had enough space. Without the restraints, the metabolized position of EPA moved slightly away from the Fe^{2+} ion (in this case, the pocket failed to be optimized for EPA). MD simulation with the restraints rearranged pocket conformations to fit the docked EPA. To verify whether the binding pockets were sufficiently optimized by the MD simulations, EPA was re-docked into the resulting structures of the MD simulation using Glide. In this second docking, bound EPA in the MD simulations was removed from the complex structures and new docked positions of EPA were determined. All water molecules around EPA at a distance of ≤ 10 Å in the MD simulation were included with the receptors. Default scaling factors for van der Waals radius (1.0 and 0.8 for the receptor and ligand, respectively) and extra precision (XP) mode with the OPLS-3 force field[7] were used. The docking grid box was placed on the center of the bound EPA in the structure of the MD simulation. The results of re-docking are shown in Supplementary Fig. S5 and S6, where EPA was bound in the almost same manner as in the final structure of the MD simulations. Consequently, it is concluded that during MD simulation, the binding pockets of CYPs were sufficiently optimized to where the same binding geometry can be predicted by a different algorithm.

References

1. Capdevila, J. H., *et al.* The highly stereoselective oxidation of polyunsaturated fatty acids by cytochrome P450BM-3. *J. Biol. Chem.* **271,** 22663–22671 (1996).
2. Catalano, J., Sadre-Bazzaz, K., Amodeo, G. A., Tong, L., & McDermott, A. Structural evidence: a single charged residue affects substrate binding in cytochrome P450 BM-3. *Biochemistry* **52,** 6807–6815 (2013).
3. Sansen, S., *et al.* Adaptations for the oxidation of polycyclic aromatic hydrocarbons

exhibited by the structure of human P450 1A2. *J. Biol. Chem.* **282,** 14348–14355 (2007).

4. Jung, C. The mystery of cytochrome P450 Compound I: a mini-review dedicated to Klaus Ruckpaul. *Biochim. Biophys. Acta* **1814,** 46–57 (2011).
5. Wills, E. D., Gillham, B., Papachristodoulou, D. K., & Thomas, J. H. Wills' biochemical basis of medicine. *Oxford: Butterworth-Heinemann* (1997).
6. Sastry, G. M., Adzhigirey, M., Day, T., Annabhimoju, R., & Sherman, W. Protein and ligand preparation: Parameters, protocols, and influence on virtual screening enrichments. *J. Comput. Aid. Mol. Des.* **27,** 221–234 (2013).
7. Harder, E., *et al.* OPLS3: A force field providing broad coverage of drug-like small molecules and proteins. *J. Chem. Theory Comput.* **12,** 281–296 (2016).
8. Friesner, R. A., *et al.* Extra precision glide: Docking and scoring incorporating a model of hydrophobic enclosure for protein-ligand complexes. *J. Med. Chem.* **49,** 6177–6196 (2006).
9. Halgren, T. A., *et al.* Glide: A new approach for rapid, accurate docking and scoring. 2. enrichment factors in database screening. *J. Med. Chem.* **47,** 1750–1759 (2004).
10. Friesner, R. A., *et al.* Glide: A new approach for rapid, accurate docking and scoring. 1. method and assessment of docking accuracy. *J. Med. Chem.* **47,** 1739–1749 (2004).
11. Farid, R., Day, T., Friesner, R. A., & Pearlstein, R. A. New insights about HERG blockade obtained from protein modeling, potential energy mapping, and docking studies. *Bioorg. & Med. Chem.* **14,** 3160–3173 (2006).
12. Sherman, W., Day, T., Jacobson, M. P., Friesner, R. A., & Farid, R. Novel procedure for modeling ligand/receptor induced fit effects. *J. Med. Chem.* **49,** 534–553 (2006).
13. Sherman, W., Beard, H. S., & Farid, R. Use of an induced fit receptor structure in virtual screening. *Chemical Biology & Drug Design* **67,** 83–84 (2006).
14. Shivakumar, D., *et al.* Prediction of absolute solvation free energies using molecular dynamics free energy perturbation and the OPLS force field. *J. Chem. Theory Comput.* **6,** 1509–1519 (2010).
15. Guo, Z., *et al.* Probing the α-helical structural stability of stapled p53 peptides: Molecular dynamics simulations and analysis. *Chem. Biol. Drug Des.* **75,** 348–359 (2010).
16. Berendsen, H. J. C., Grigera, J. R., & Straatsma, T. P. The missing term in effective pair potentials. *J. Phys. Chem.* **91,** 6269–6271 (1987).
17. Evans, D. J., & Holian, B. L. The nose-hoover thermostat. *J. Chem. Phys.* **83,** 4069–4074 (1985).
18. Martyna, G. J. Remarks on Constant-temperature molecular dynamics with momentum

conservation. Phys. Rev. E Stat. Phys. Plasmas Fluids Relat. Interdiscip Topics **50,** 3234–3236 (1994).

19. Martyna, G. J., Klein, M. L., & Tuckerman, M. Nose–Hoover chains: the canonical ensemble via continuous dynamics. *J. Chem. Phys.* **97,** 2635–2643 (1992).
20. Essmann, E., *et al.* A smooth particle mesh Ewald method. *J. Chem. Phys.* **103,** 8577–8593 (1995).
21. Tuckerman, M., Berne, B. J., & Martyna, G. J. Reversible multiple time scale molecular dynamics. *J. Chem. Phys.* **97,** 1990–2001 (1992).
22. Ryckaert, J-P., Ciccotti, G., & Berendsen, H. J. C. Numerical integration of the cartesian equations of motion of a system with constraints: molecular dynamics of n-alkanes. *J. Comp. Phys.* **23,** 327–341 (1977).

Supplementary Table S1

Table. S1. Metabolisms of AA, EPA, and DHA by Cyp1a2-transfected cells.

Amounts of each metabolite formed from AA, EPA and DHA by Cyp1a2-transfected cells were determined by subtracting background levels measured in mock-transfected cells. Values represent the mean ± SEM; n = 3. ND, not detectable.

AA		EPA		DHA	
metabolite	amount (ng/10^5 cells)	metabolite	amount (ng/10^5 cells)	metabolite	amount (ng/10^5 cells)
5-HETE	ND	5-HEPE	0.01±0.27	4-HDoHE	ND
5,6-EET	1.13±0.17	5,6-diHETE	ND	7-HDoHE	ND
5,6-DHT	0.01±0.002	8-HEPE	ND	8-HDoHE	ND
8-HETE	ND	9-HEPE	ND	7,8-EpDPE	0.03±0.01
9-HETE	ND	8,9-EpETE	0.15±0.02	7,8-diHDoPE	ND
8,9-EET	0.05±0.01	8,9-diHETE	ND	10-HDoHE	ND
8,9-DHT	0.01±0.001	11-HEPE	ND	11-HDoHE	ND
11-HETE	ND	12-HEPE	ND	10,11-EpDPE	ND
12-HETE	ND	11,12-EpETE	0.27±0.02	10,11-diHDoPE	0.01±0.001
11,12-EET	0.06±0.01	11,12-diHETE	0.17±0.003	13-HDoHE	ND
11,12-DHT	0.04±0.005	15-HEPE	0.06±0.09	14-HDoHE	ND
15-HETE	ND	14,15-EpETE	0.15±0.02	13,14-EpDPE	0.05±0.01
14,15-EET	0.15±0.01	14,15-diHETE	0.71±0.09	13,14-diHDoPE	0.06±0.01
14,15-DHT	0.11±0.01	18-HEPE	ND	16-HDoHE	ND
16-HETE	0.38±0.02	17,18-EpETE	14.28±1.81	17-HDoHE	ND
17-HETE	0.43±0.03	17,18-diHETE	3.17±0.19	16,17-EpDPE	ND
18-HETE	0.55±0.02	19-HEPE	1.95±0.34	16,17-diHDoPE	ND
19-HETE	0.64±0.02	20-HEPE	ND	20-HDoHE	ND
20-HETE	ND			19,20-EpDPE	2.65±0.12
				19,20-diHDoPE	0.61±0.03
				21-HDoHE	0.26±0.02
				22-HDoHE	ND

Supplementary Table S2

Table. S2. Metabolisms of AA, EPA, and DHA by Cyp2c50-transfected cells.
Amounts of each metabolite formed from AA, EPA and DHA by Cyp2c50-transfected cells were determined by subtracting background levels measured in mock-transfected cells. Values represent the mean ± SEM; n = 3. ND, not detectable.

AA		EPA		DHA	
metabolite	amount (ng/10^5 cells)	metabolite	amount (ng/10^5 cells)	metabolite	amount (ng/10^5 cells)
5-HETE	0.14±0.18	5-HEPE	ND	4-HDoHE	ND
5,6-EET	4.75±0.43	5,6-diHETE	2.56±1.28	7-HDoHE	ND
5,6-DHT	0.03±0.002	8-HEPE	0.12±0.19	8-HDoHE	ND
8-HETE	0.10±0.09	9-HEPE	ND	7,8-EpDPE	0.24±0.02
9-HETE	0.06±0.07	8,9-EpETE	2.72±0.33	7,8-diHDoPE	0.02±0.02
8,9-EET	0.60±0.05	8,9-diHETE	0.19±0.01	10-HDoHE	ND
8,9-DHT	0.16±0.02	11-HEPE	0.13±0.17	11-HDoHE	ND
11-HETE	0.10±0.04	12-HEPE	ND	10,11-EpDPE	0.18±0.02
12-HETE	0.06±0.08	11,12-EpETE	3.83±0.37	10,11-diHDoPE	0.03±0.002
11,12-EET	1.32±0.08	11,12-diHETE	0.85±0.05	13-HDoHE	ND
11,12-DHT	0.31±0.02	15-HEPE	0.06±0.12	14-HDoHE	ND
15-HETE	0.09±0.18	14,15-EpETE	2.18±0.17	!3,14-EpDPE	0.21±0.01
14,15-EET	0.88±0.05	14,15-diHETE	6.57±0.43	13,14-diHDoPE	0.14±0.02
14,15-DHT	0.58±0.03	18-HEPE	ND	16-HDoHE	ND
16-HETE	0.09±0.02	17,18-EpETE	4.16±0.39	17-HDoHE	ND
17-HETE	0.07±0.01	17,18-diHETE	0.62±0.07	16,17-EpDPE	0.10±0.01
18-HETE	0.001±0.01	19-HEPE	2.54±0.37	16,17-diHDoPE	0.02±0.003
19-HETE	0.13±0.02	20-HEPE	ND	20-HDoHE	ND
20-HETE	ND			19,20-EpDPE	0.11±0.01
				19,20-diHDoPE	0.02±0.01
				21-HDoHE	0.31±0.01
				22-HDoHE	ND

Supplementary Table S3

Table. S3. Metabolisms of AA, EPA, and DHA by Cyp4a12a-transfected cells.
Amounts of each metabolite formed from AA, EPA and DHA by Cyp4a12a-transfected cells were determined by subtracting background levels measured in mock-transfected cells. Values represent the mean ± SEM; n = 3. ND, not detectable.

AA		EPA		DHA	
metabolite	amount (ng/10^5 cells)	metabolite	amount (ng/10^5 cells)	metabolite	amount (ng/10^5 cells)
5-HETE	ND	5-HEPE	ND	4-HDoHE	ND
5,6-EET	ND	5,6-diHETE	ND	7-HDoHE	ND
5,6-DHT	ND	8-HEPE	ND	8-HDoHE	ND
8-HETE	ND	9-HEPE	ND	7,8-EpDPE	ND
9-HETE	ND	8,9-EpETE	ND	7,8-diHDoPE	ND
8,9-EET	ND	8,9-diHETE	ND	10-HDoHE	ND
8,9-DHT	ND	11-HEPE	ND	11-HDoHE	ND
11-HETE	ND	12-HEPE	ND	10,11-EpDPE	ND
12-HETE	ND	11,12-EpETE	ND	10,11-diHDoPE	0.002±0.002
11,12-EET	ND	11,12-diHETE	ND	13-HDoHE	ND
11,12-DHT	ND	15-HEPE	ND	14-HDoHE	ND
15-HETE	ND	14,15-EpETE	ND	13,14-EpDPE	0.01±0.01
14,15-EET	ND	14,15-diHETE	0.08±0.01	13,14-diHDoPE	ND
14,15-DHT	ND	18-HEPE	1.97±0.64	16-HDoHE	0.65±0.20
16-HETE	ND	17,18-EpETE	41.79±1.68	17-HDoHE	ND
17-HETE	0.06±0.01	17,18-diHETE	10.88±1.40	16,17-EpDPE	ND
18-HETE	0.39±0.04	19-HEPE	33.38±1.11	16,17-diHDoPE	0.001±0.01
19-HETE	2.40±0.36	20-HEPE	17.13±0.88	20-HDoHE	2.20±0.60
20-HETE	21.03±1.82			19,20-EpDPE	11.25±2.86
				19,20-diHDoPE	1.77±0.32
				21-HDoHE	3.63±0.59
				22-HDoHE	4.32±0.72

Supplementary Table S4

Table. S4. Metabolisms of AA, EPA, and DHA by Cyp4a12b-transfected cells.

Amounts of each metabolite formed from AA, EPA and DHA by Cyp4a12b-transfected cells were determined by subtracting background levels measured in mock-transfected cells. Values represent the mean ± SEM; n = 3. ND, not detectable.

AA		EPA		DHA	
metabolite	amount (ng/10^5 cells)	metabolite	amount (ng/10^5 cells)	metabolite	amount (ng/10^5 cells)
5-HETE	ND	5-HEPE	ND	4-HDoHE	ND
5,6-EET	ND	5,6-diHETE	ND	7-HDoHE	ND
5,6-DHT	ND	8-HEPE	ND	8-HDoHE	ND
8-HETE	ND	9-HEPE	ND	7,8-EpDPE	ND
9-HETE	ND	8,9-EpETE	ND	7,8-diHDoPE	ND
8,9-EET	0.003±0,003	8,9-diHETE	ND	10-HDoHE	ND
8,9-DHT	ND	11-HEPE	ND	11-HDoHE	ND
11-HETE	ND	12-HEPE	ND	10,11-EpDPE	ND
12-HETE	ND	11,12-EpETE	ND	10,11-diHDoPE	ND
11,12-EET	ND	11,12-diHETE	ND	13-HDoHE	ND
11,12-DHT	ND	15-HEPE	ND	14-HDoHE	ND
15-HETE	ND	14,15-EpETE	0.05±0.01	13,14-EpDPE	0.03±0.02
14,15-EET	0.01±0.002	14,15-diHETE	0.15±0.02	13,14-diHDoPE	ND
14,15-DHT	0.01±0.002	18-HEPE	ND	16-HDoHE	ND
16-HETE	ND	17,18-EpETE	9.32±0.97	17-HDoHE	ND
17-HETE	0.02±0.004	17,18-diHETE	2.57±0.47	16,17-EpDPE	ND
18-HETE	0.38±0.04	19-HEPE	0.91±0.07	16,17-diHDoPE	ND
19-HETE	0.27±0.05	20-HEPE	0.63±0.02	20-HDoHE	ND
20-HETE	3.48±0.41			19,20-EpDPE	0.46±0.12
				19,20-diHDoPE	0.12±0.01
				21-HDoHE	0.16±0.02
				22-HDoHE	0.06±0.005

Supplementary Table S5

Table. S5. Metabolisms of AA, EPA, and DHA by Cyp4f18-transfected cells.
Amounts of each metabolite formed from AA, EPA and DHA by Cyp4f18-transfected cells were determined by subtracting background levels measured in mock-transfected cells. Values represent the mean ± SEM; n = 3. ND, not detectable.

AA		EPA		DHA	
metabolite	amount (ng/10^5 cells)	metabolite	amount (ng/10^5 cells)	metabolite	amount (ng/10^5 cells)
5-HETE	ND	5-HEPE	ND	4-HDoHE	ND
5,6-EET	0.03±0.03	5,6-diHETE	ND	7-HDoHE	ND
5,6-DHT	ND	8-HEPE	ND	8-HDoHE	ND
8-HETE	ND	9-HEPE	ND	7,8-EpDPE	ND
9-HETE	ND	8,9-EpETE	ND	7,8-diHDoPE	ND
8,9-EET	ND	8,9-diHETE	ND	10-HDoHE	ND
8,9-DHT	ND	11-HEPE	ND	11-HDoHE	ND
11-HETE	ND	12-HEPE	ND	10,11-EpDPE	ND
12-HETE	ND	11,12-EpETE	0.01±0.01	10,11-diHDoPE	ND
11,12-EET	ND	11,12-diHETE	ND	13-HDoHE	ND
11,12-DHT	ND	15-HEPE	ND	14-HDoHE	ND
15-HETE	ND	14,15-EpETE	ND	13,14-EpDPE	ND
14,15-EET	ND	14,15-diHETE	ND	13,14-diHDoPE	ND
14,15-DHT	ND	18-HEPE	ND	16-HDoHE	ND
16-HETE	ND	17,18-EpETE	17.88±1.05	17-HDoHE	ND
17-HETE	0.26±0.02	17,18-diHETE	5.86±0.87	16,17-EpDPE	ND
18-HETE	14.87±1.54	19-HEPE	24.15±2.20	16,17-diHDoPE	ND
19-HETE	8.63±1.07	20-HEPE	0.08±0.01	20-HDoHE	0.15±0.07
20-HETE	1.01±0.09			19,20-EpDPE	30.20±3.30
				19,20-diHDoPE	3.61±0.19
				21-HDoHE	9.87±0.83
				22-HDoHE	ND

Supplementary Table S6

Table. S6. MRM transitions (Q1 and Q3), declustering potential (DP), entrance potential (EP), collision energy (CE), collision cell exit potential (CXP), and retention time (RT) for all the analytes and internal standards used in this study.

analyte	Q1	Q3	DP	EP	CE	CXP	RT (min)
5-HETE	319.01	114.9	-65	-10	-20	-11	22.5
5,6-EET	319.192	191.1	-70	-10	-16	-13	24.9
5,6-DHT	337.204	144.8	-65	-10	-22	-14	20.0
8-HETE	319.177	154.9	-90	-10	-18	-13	21.7
9-HETE	319.185	123	-110	-10	-22	-17	22.1
8,9-EET	319.188	154.9	-35	-10	-16	-11	24.3
8,9-DHT	337.195	126.9	-55	-10	-28	-9	19.3
11-HETE	319.171	166.9	-70	-10	-20	-13	21.3
12-HETE	319.156	179	-25	-10	-18	-11	21.6
11,12-EET	319.179	167	-75	-10	-18	-17	23.9
11,12-DHT	337.172	167	-70	-10	-26	-7	18.6
15-HETE	319.156	219	-50	-10	-20	-15	20.8
14,15-EET	319.181	218.9	-65	-10	-16	-15	23.0
14,15-DHT	337.183	207.1	-60	-10	-24	-19	18.3
16-HETE	319.165	233.2	-70	-10	-18	-11	20.1
17-HETE	319.16	247	-75	-10	-20	-15	20.0
18-HETE	319.193	261.2	-90	-10	-22	-21	19.8
19-HETE	319.159	275	-75	-10	-24	-15	19.5
20-HETE	319.171	289.1	-120	-10	-24	-27	19.6
5-HEPE	317.12	115	-70	-10	-18	-17	20.4
5,6-diHETE	335.1	189	-95	-10	-22	-17	18.4
8-HEPE	317.126	154.9	-85	-10	-20	-15	19.8
9-HEPE	317.147	148.9	-50	-10	-22	-7	20.1
8,9-EpETE	317.161	155.1	-85	-10	-14	-19	21.8
8,9-diHETE	335.041	127	-120	-10	-24	-11	17.7
11-HEPE	317.179	167	-85	-10	-22	-11	19.6
12-HEPE	317.158	179	-60	-10	-18	-13	19.8
11,12-EpETE	317.149	166.9	-85	-10	-16	-11	21.5
11,12-diHETE	335.046	167.1	-115	-10	-24	-11	17.4
15-HEPE	317.159	219	-45	-10	-18	-19	19.5
14,15-EpETE	317.232	207.1	-90	-10	-16	-15	21.4

analyte	Q1	Q3	DP	EP	CE	CXP	RT (min)
14,15-diHETE	335.1	207.1	-105	-10	-24	-19	17.3
18-HEPE	317.188	259.3	-40	-10	-16	-9	19.1
17,18-EpETE	317.192	258.9	-55	-10	-16	-29	20.8
17,18-diHETE	335.165	246.9	-65	-10	-24	-23	17.0
19-HEPE	317.146	229.2	-50	-10	-20	-21	18.8
20-HEPE	317.19	287.1	-60	-10	-20	-9	18.8
4-HDoHE	343.167	100.9	-40	-10	-16	-35	22.9
7-HDoHE	343.193	141.1	-55	-10	-14	-13	21.8
8-HDoHE	343.224	189	-55	-10	-16	-11	22.0
7,8-EpDPE	343.182	109	-40	-10	-18	-13	24.0
7,8-diHDoPE	361.09	113	-90	-10	-24	-5	19.5
10-HDoHE	343.202	153	-70	-10	-24	-21	21.3
11-HDoHE	343.18	121.1	-80	-10	-20	-13	21.6
10,11-EpDPE	343.189	153	-40	-10	-16	-15	23.54
10,11-diHDoPE	361.089	153	-95	-10	-24	-17	18.9
13-HDoHE	343.185	193	-40	-10	-18	-15	21.01
14-HDoHE	343.206	205	-50	-10	-18	-17	21.22
13,14-EpDPE	343.189	193	-40	-10	-16	-13	23.3
13,14-diHDoPE	361.09	193	-105	-10	-24	-11	18.7
16-HDoHE	343.164	233.1	-65	-10	-20	-9	20.8
17-HDoHE	343.194	245.1	-35	-10	-16	-21	20.9
16,17-EpDPE	343.213	233	-50	-10	-16	-9	23.2
16,17-diHDoPE	361.091	233.2	-100	-10	-22	-9	18.5
20-HDoHE	343.192	241.1	-45	-10	-18	-27	20.4
19,20-EpDPE	343.207	241.1	-55	-10	-18	-21	22.6
19,20-diHDoPE	361.194	229.3	-30	-10	-24	-21	18.3
21-HDoHE	343.184	255	-65	-10	-18	-19	20.1
22-HDoHE	343.167	269.1	-65	-10	-20	-17	20.2
15-HETE-d8	327.106	226.2	-70	-10	-18	-19	20.6
14,15-EET-d11	330.2	218.9	-65	-10	-16	-15	22.8
LTB_4-d4	339.196	197.2	-60	-10	-22	-13	17.2

Supplementary Figure S1

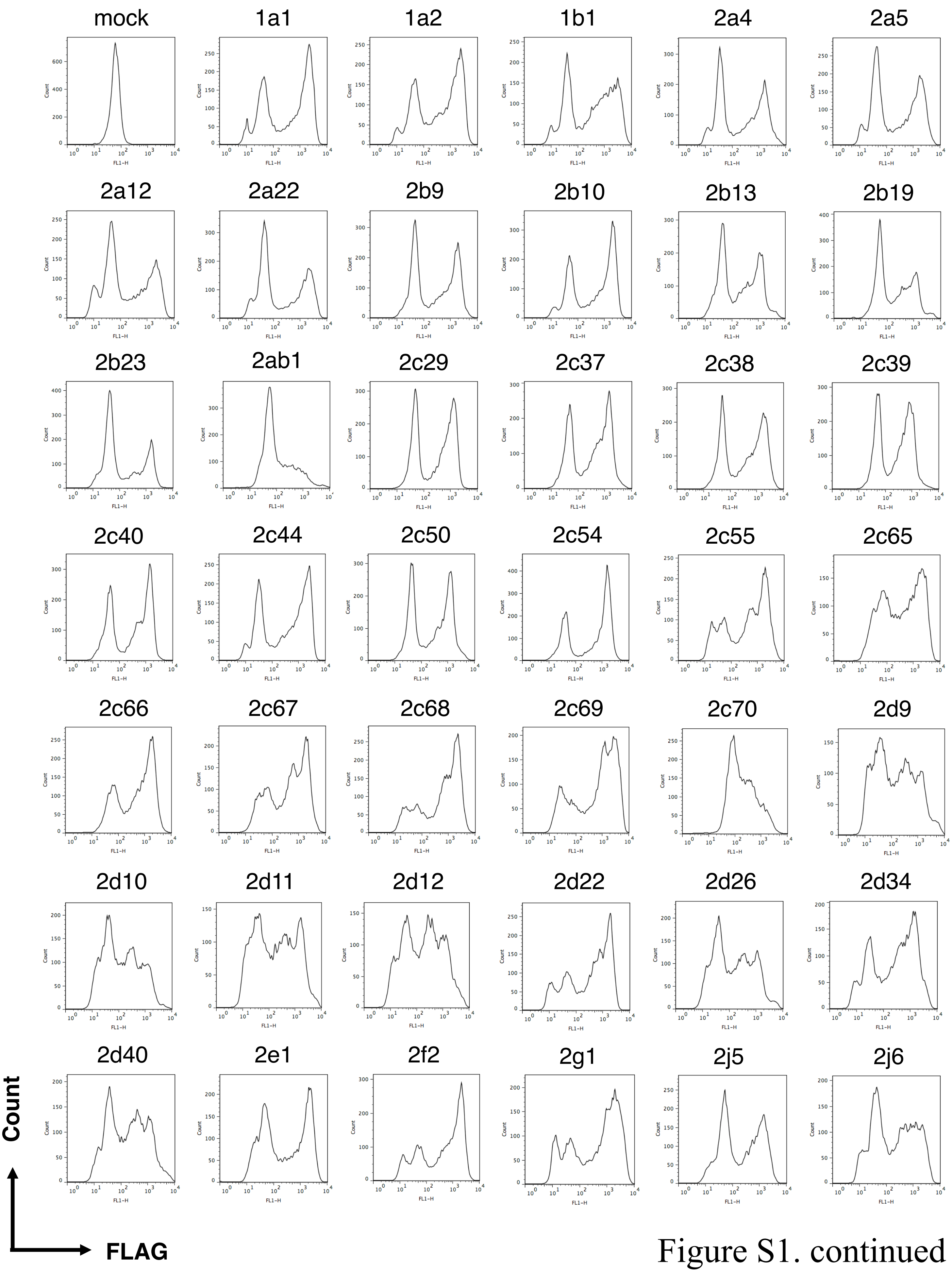

Figure S1. continued

Supplementary Figure S1

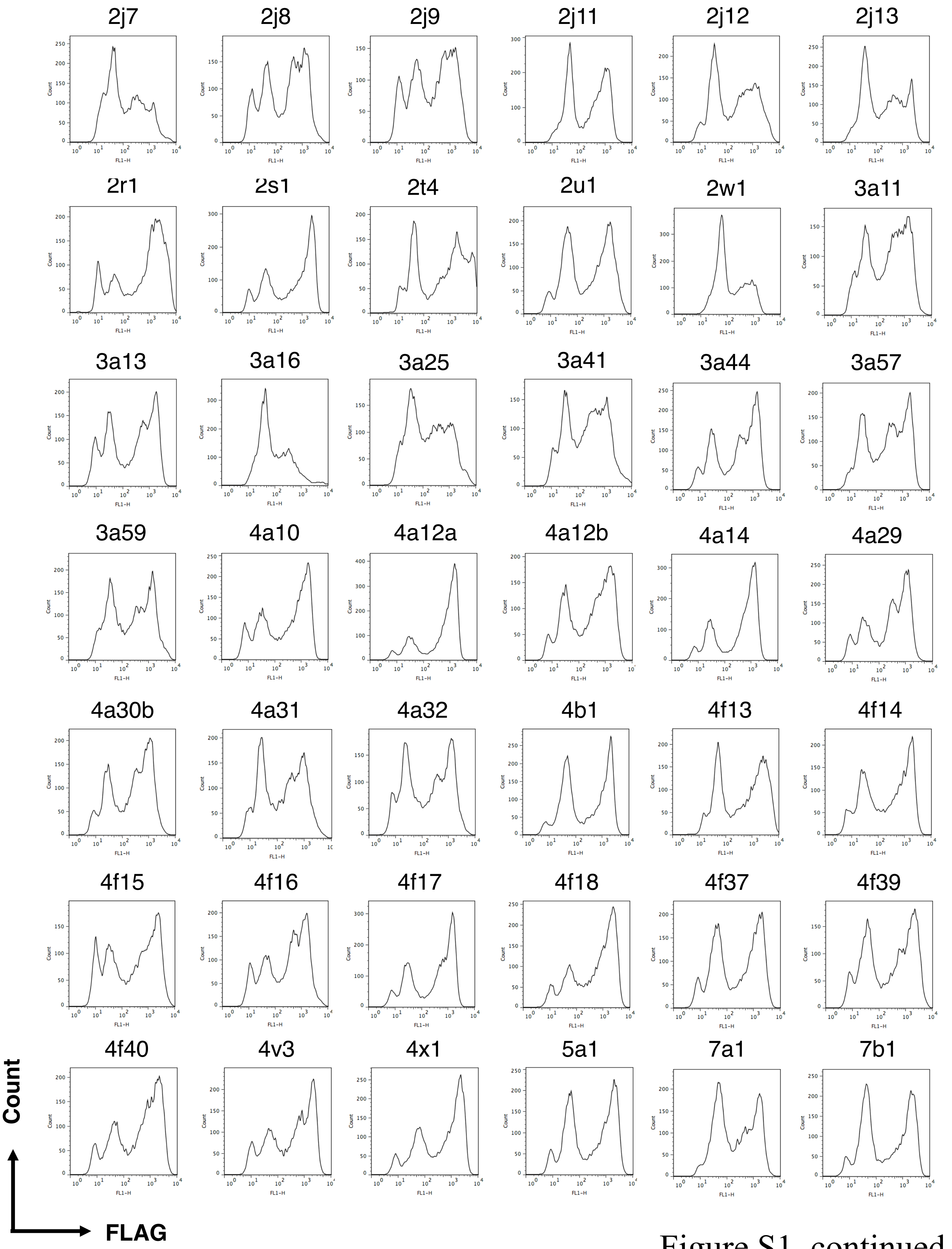

Figure S1. continued

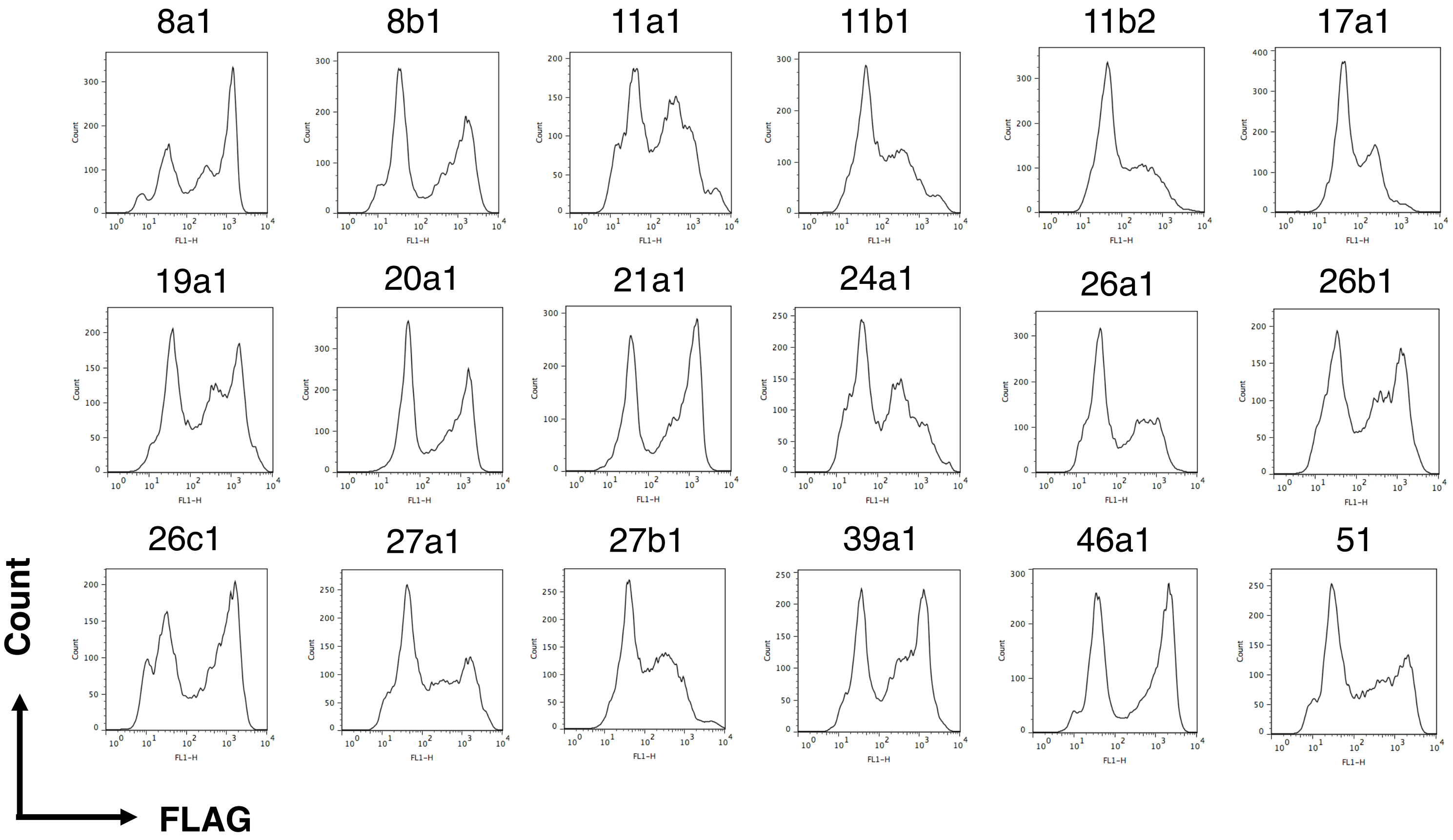

Figure S1. Protein expression of CYP enzymes tested in this study. Shown is flow cytometry analysis of HEK293 cells expressing FLAG-tagged CYP isoforms. Cells were stained with anti-FLAG antibody (L5), followed by staining with Alexa Fluor 488–conjugated anti-rat IgG.

Supplementary Figure S2

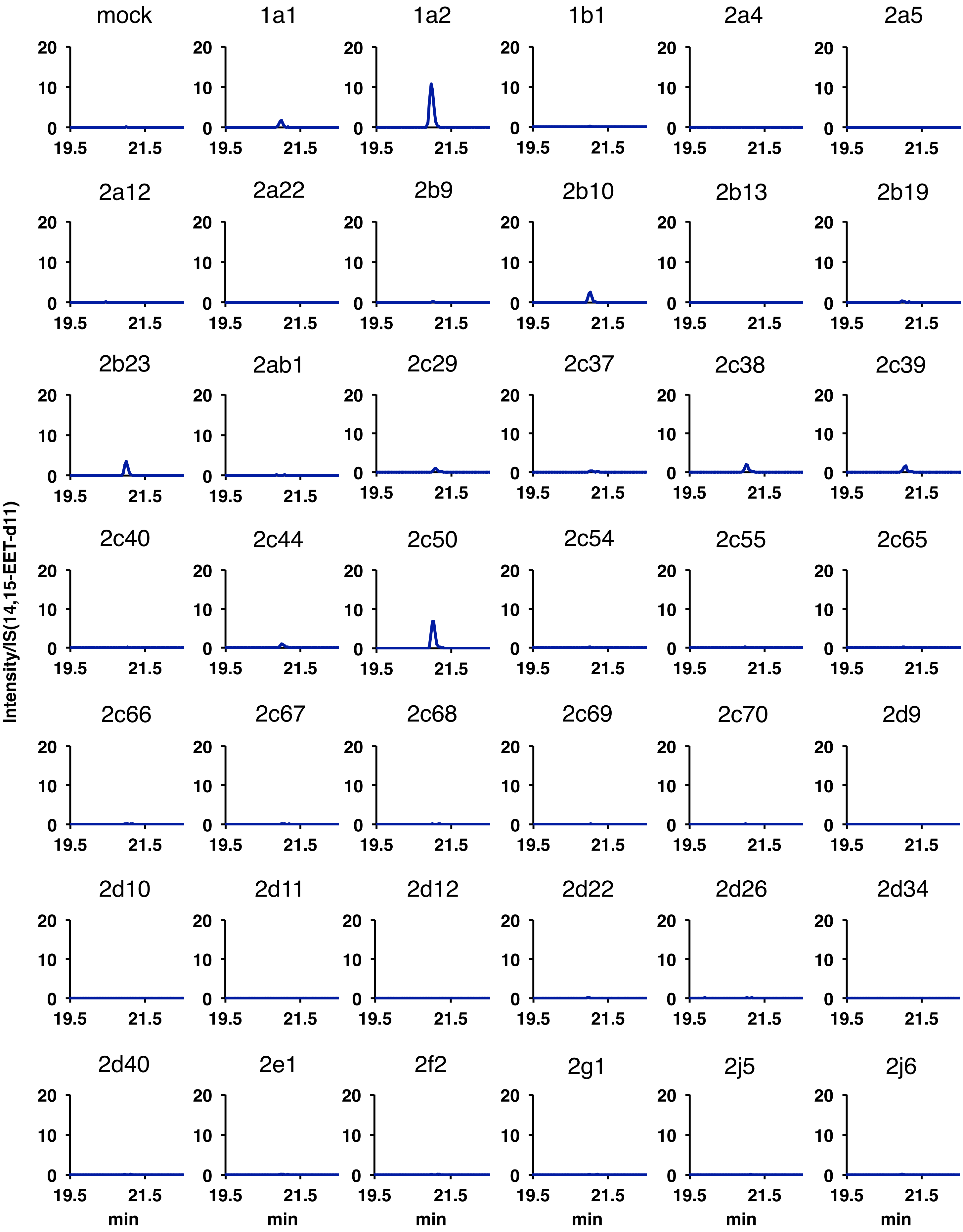

Figure S2. continued

Supplementary Figure S2

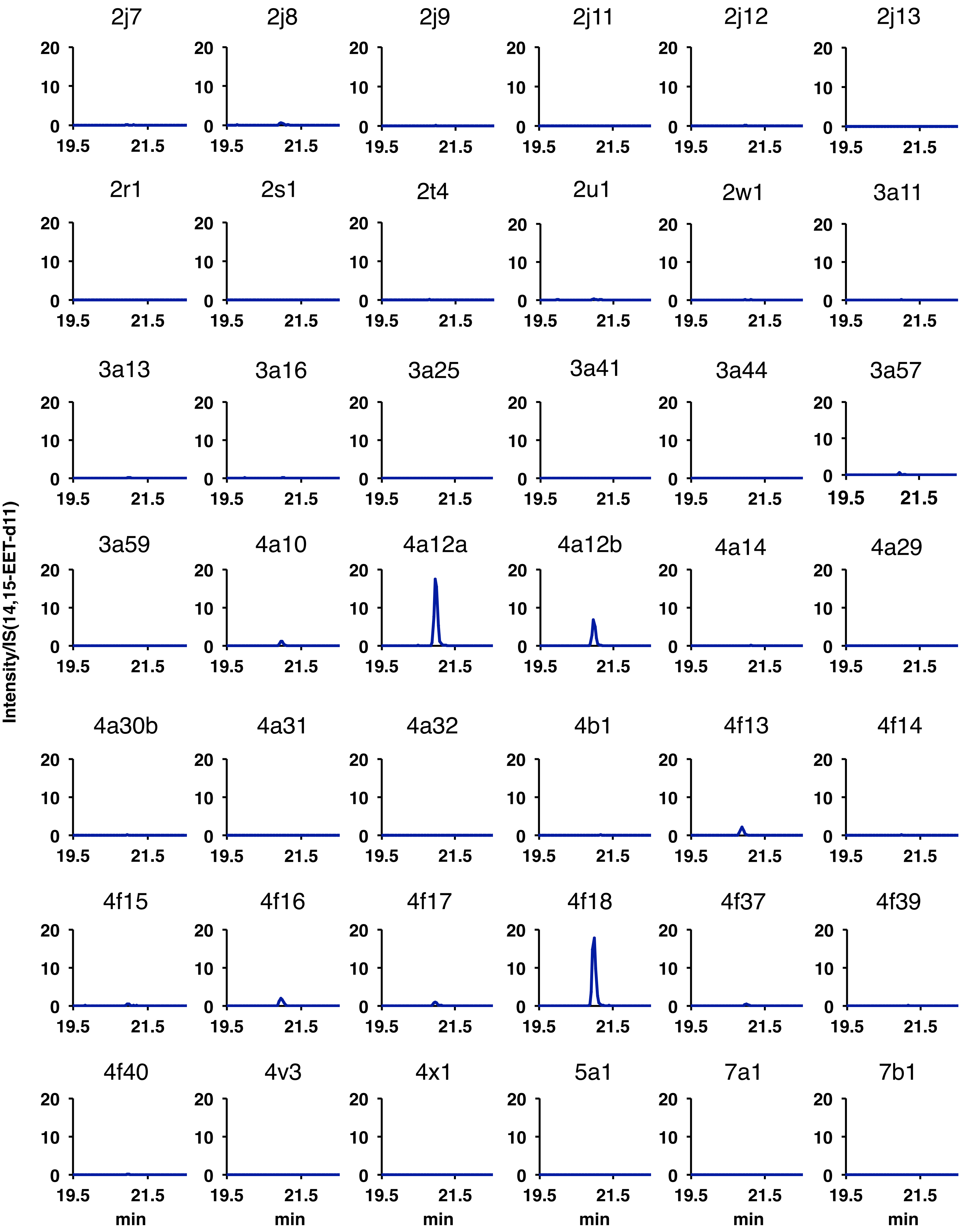

Figure S2. continued

Supplementary Figure S2

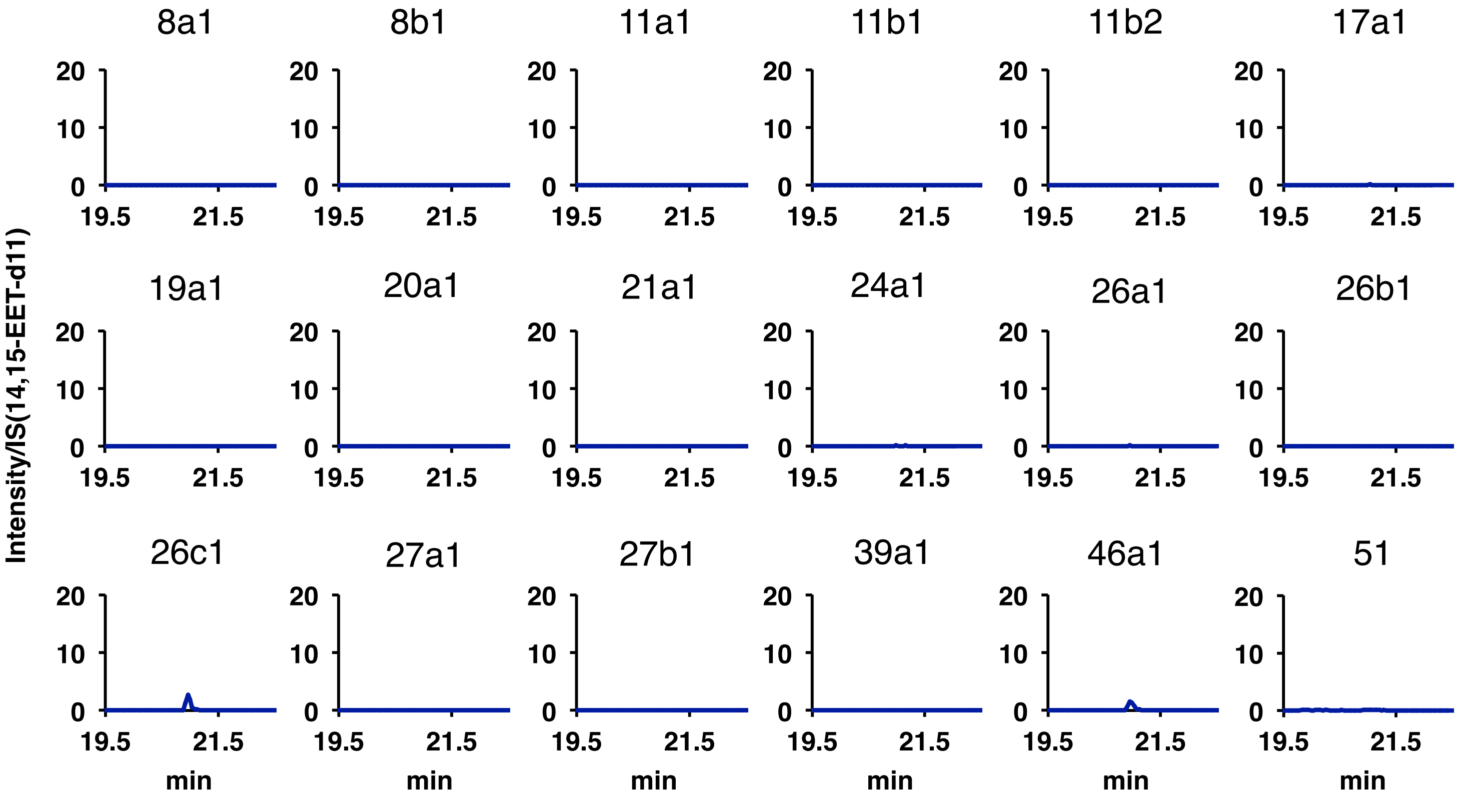

Figure S2. MRM chromatograms of 17,18-EpETE which are produced by HEK293 cells transiently transfected with the mouse CYP cDNA library.

Supplementary Figure S3

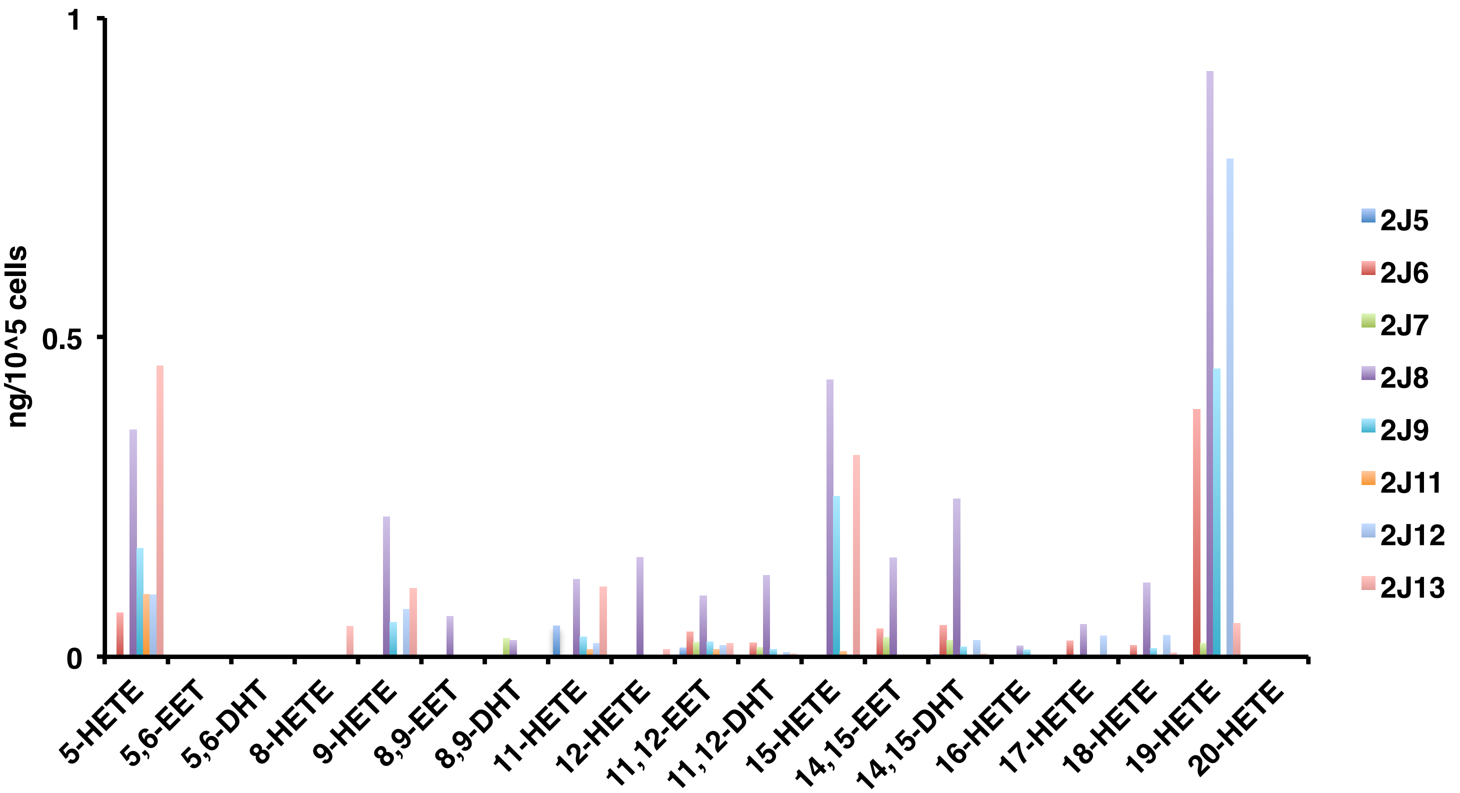

Figure S3. Product profiles in AA metabolism by cells expressing mouse Cyp2j enzymes.

Supplementary Figure S4

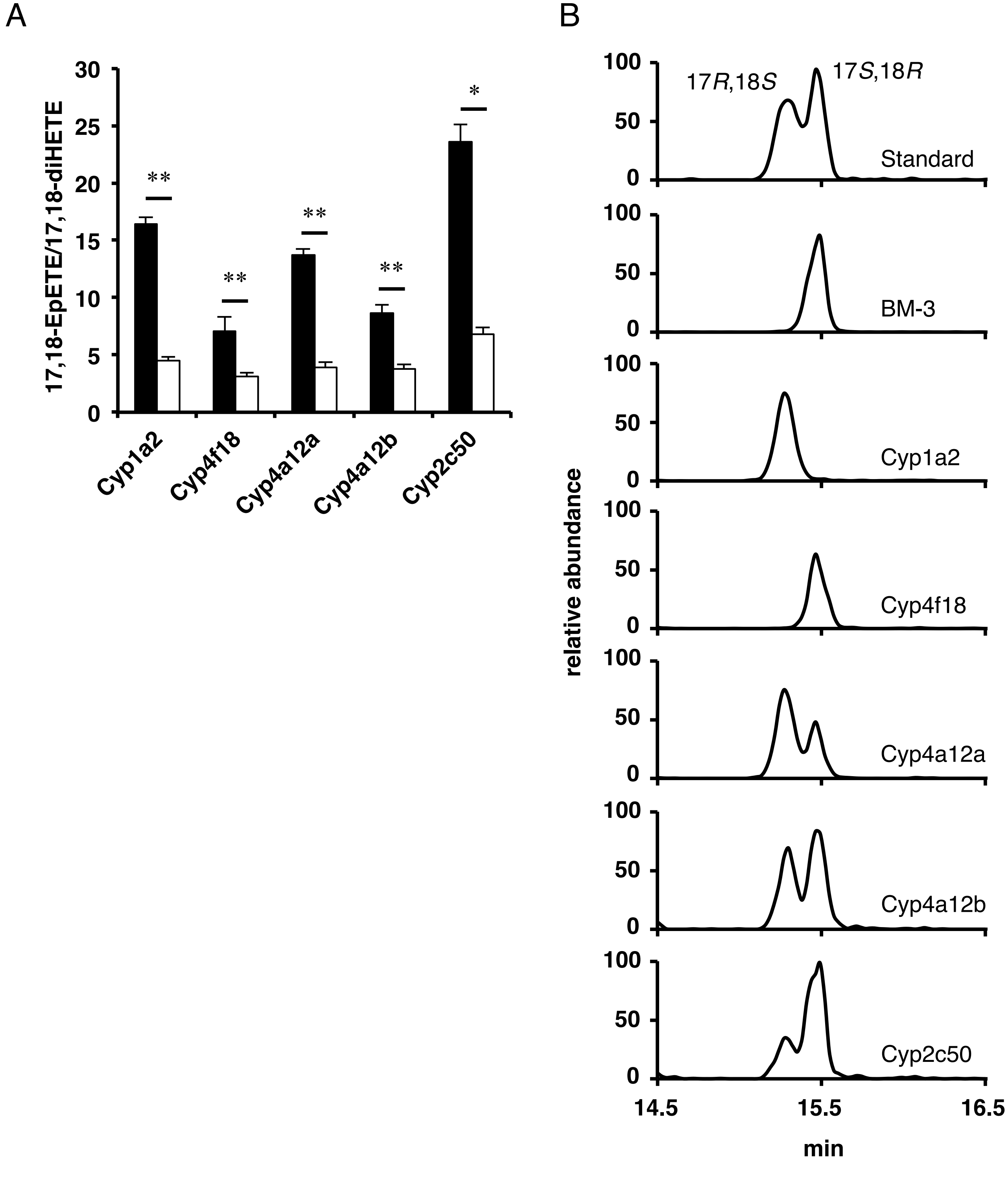

Figure S4. Stereochemistry of omega-3 epoxidation of EPA by candidate CYP isoforms in the presence of sEH inhibitor. (A) HEK293 cells expressing each CYP enzyme were incubated with EPA in the presence (20µM, black bar) or absence (white bar) of sEH inhibitor, CUDA. The ratio of 17,18-EpETE/17,18-diHETE were calculated. Values represent the mean ± SEM; n = 3. *P< 0.05, **P< 0.01.

Supplementary Figure S5

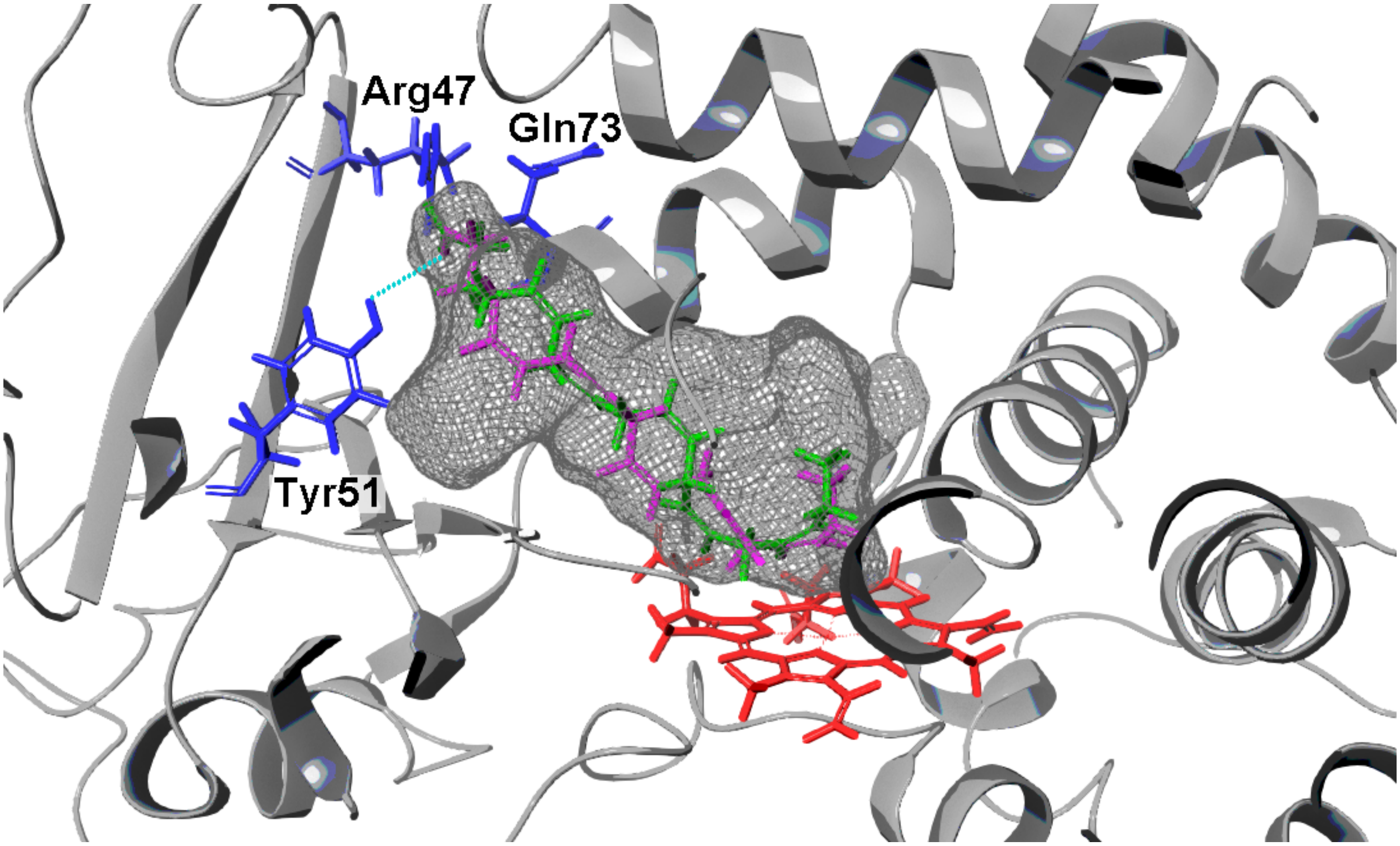

Figure S5. Bound EPA in the last snapshot of MD simulation of the BM-3 structure is shown in green. Docked EPA in the same structure using Glide is shown in magenta. Heme is indicated in red, and the three most interacted residues with EPA (Arg47, Tyr51, and Gln73) are shown in blue. The binding pocket calculated by SiteMap (Schrodinger, LLC) is showed in mesh.

Supplementary Figure S6

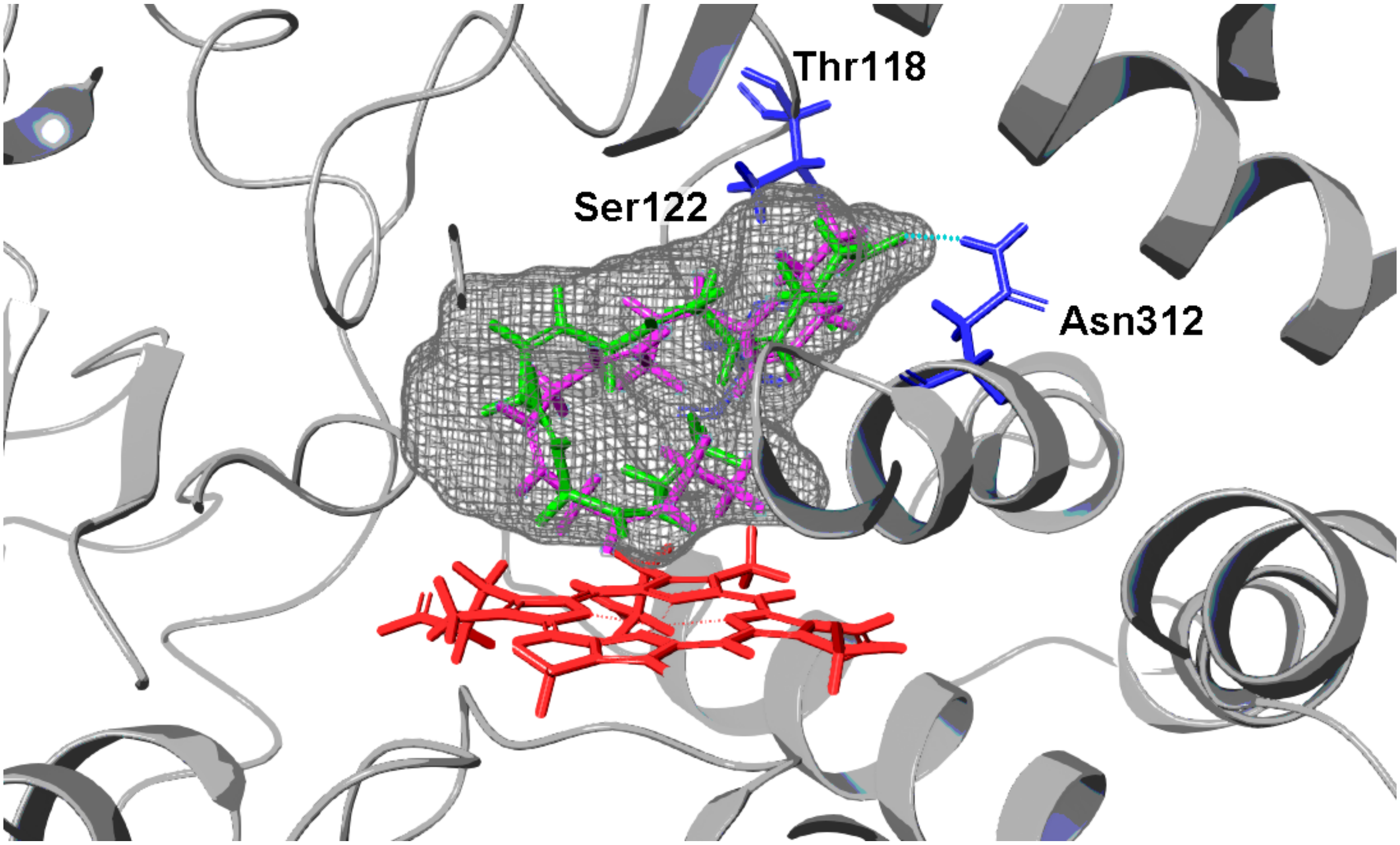

Figure S6. Bound EPA in the last snapshot of MD simulation of human CYP1A2 is shown in green. Docked EPA in the same structure using Glide is shown in magenta. Heme is indicated in red, and the three most interacted residues with EPA (Thr118, Ser122, and Asn312) are shown in blue. Located behind the binding cavity, Ser122 is hidden by the surface pocket. The binding pocket calculated by SiteMap (Schrodinger, LLC) is shown in mesh.

Supplementary Figure S7

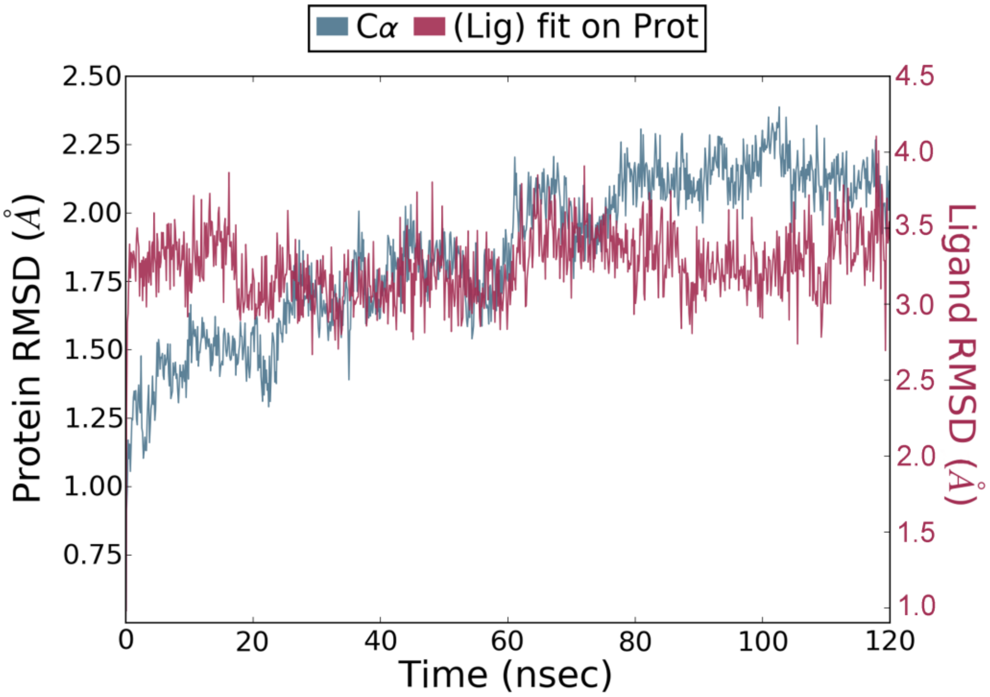

Figure S7. RMSD value for the Cα atoms of BM-3 (blue) and for the EPA (red) during the MD simulation. The reference is the EPA and BM-3 complex predicted by Glide.

Supplementary Figure S8

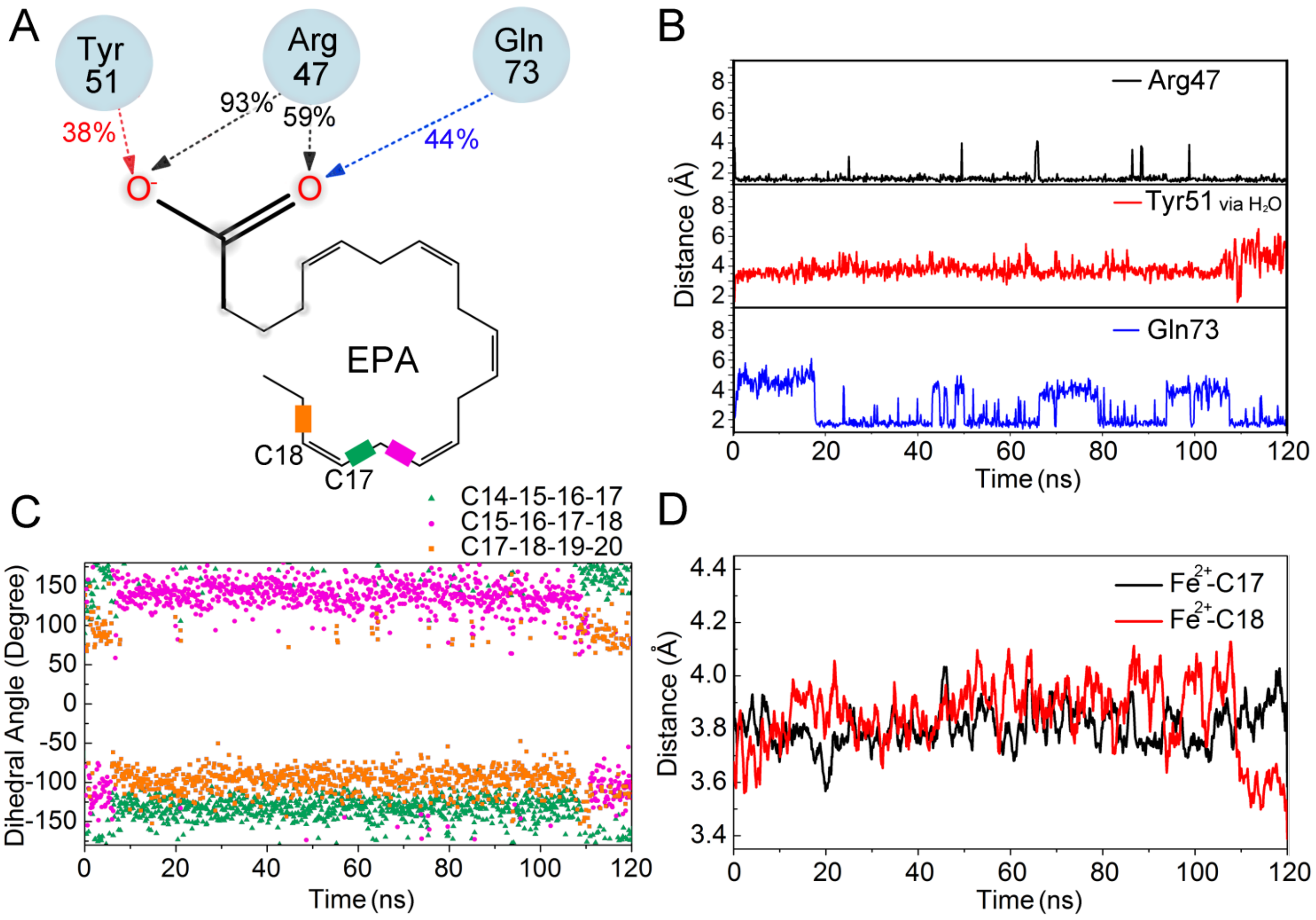

Figure S8. MD simulation of BM-3. (A) The interaction summary between residues and EPA in MD simulation of BM-3. The three most interacted residues with EPA are shown with interaction percentages. Interaction criterion is the distance less than 2.5 Å between the residue and EPA (if the residues have a hydrogen bond via a water atom, the criteria is less than 2.7 Å). (B) Interactions of the three residues shown in (A) are monitored during the 120 ns MD simulation. The colors are matched to (A). (C) The dihedral angles around the metabolized position (C17 and 18) during MD simulation are shown. Measured angles are indicated in (A) by rectangles with colors matched to the colored dots in (C). (D) The distance between the metabolized position and Fe^{2+}. (C) and (D) indicate that during most MD simulations, EPA is bound in the conformation metabolized to 17*S*,18*R*-EpETE.

Supplementary Figure S9

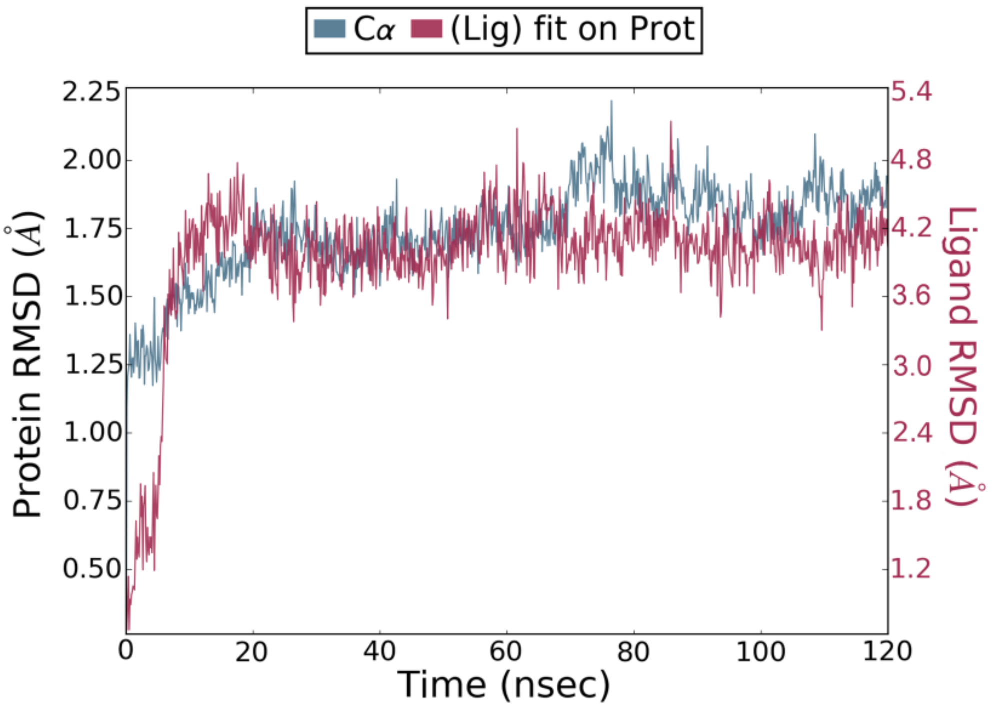

Figure S9. RMSD value for the Cα atoms of human CYP1A2 (blue) and for the EPA (red) during the MD simulation. The reference is the EPA and CYP1A2 complex predicted by Glide.

Supplementary Figure S10

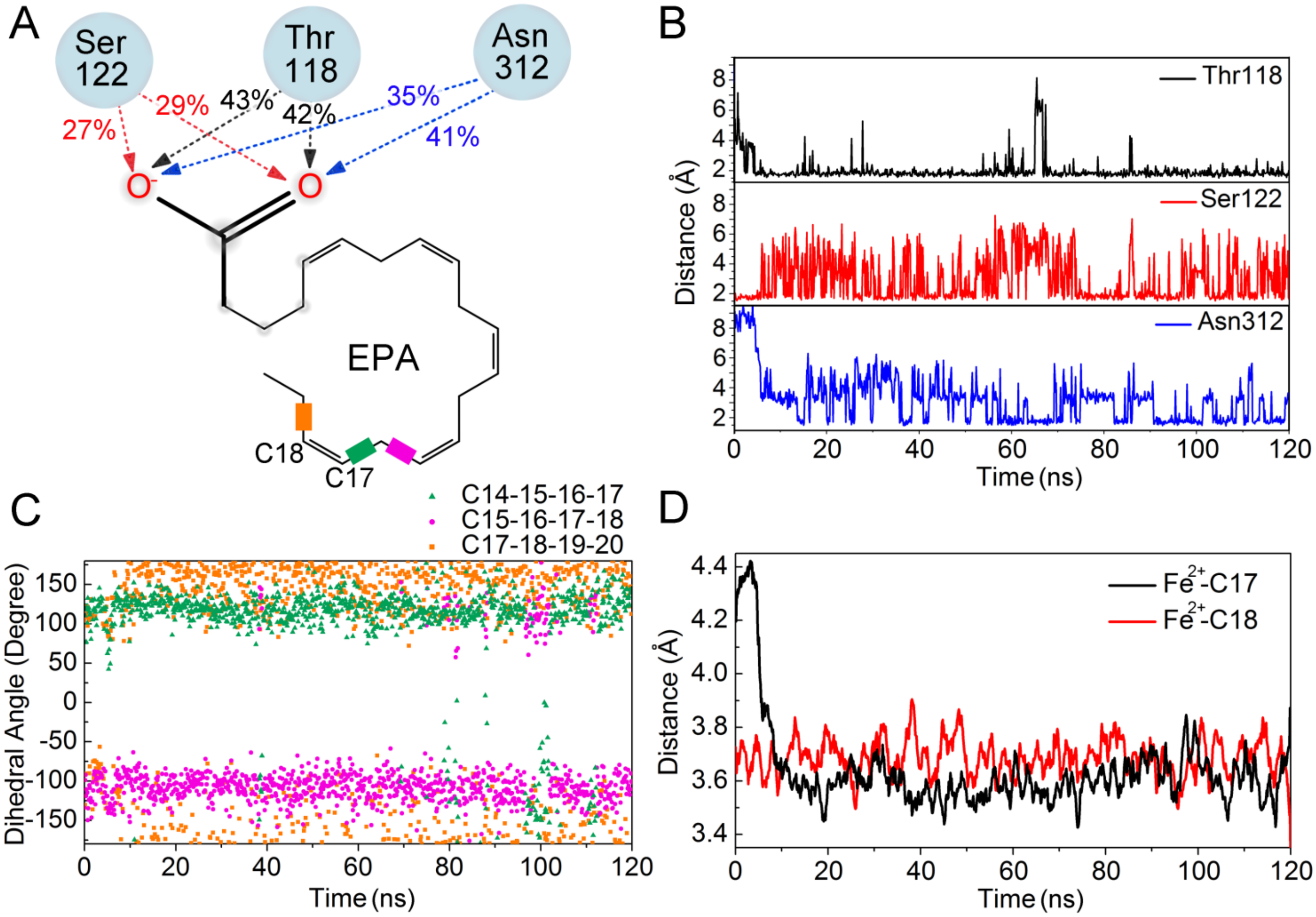

Figure S10. MD simulation of human CYP1A2. (A) Interaction summary between residues and EPA in MD simulation of human CYP1A2. The three most interacted residues with EPA are shown with interaction percentages. Interaction criterion is described in Figure S1. (B) Interactions of the three residues shown in (A) are monitored during the 120 ns MD simulation. The colors are matched to (A). (C) Dihedral angles around the metabolized position (C17 and 18) during MD simulation are shown. Measured angles are indicated in (A) by rectangles whose colors are matched to the colored dots in (C). (D) The distance between the metabolized position and Fe^{2+}. (C) and (D) indicate that during the most MD simulations, EPA is bound in the conformation metabolized to 17*R*,18*S*-EpETE.

Supplementary Figure S11

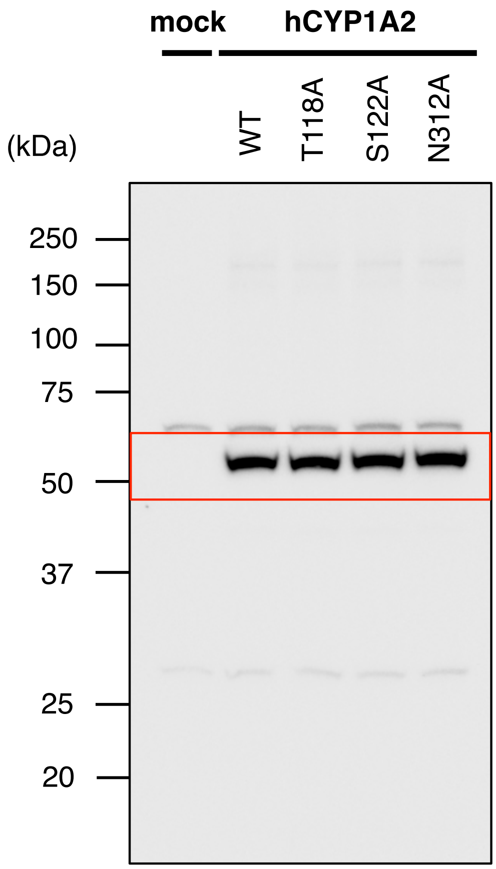

Figure S11. The original western blotting image used in Fig. 4E.
